# Anti-Alzheimer’s Natural Products Derived from Plant Endophytic Fungi

**DOI:** 10.3390/molecules28052259

**Published:** 2023-02-28

**Authors:** Juntai Zhu, Zimo Wang, Lixia Song, Wanxin Fu, Li Liu

**Affiliations:** 1 Institute of Chinese Materia Medica, China Academy of Chinese Medical Sciences, Beijing 100700, China; 2 Center for Medical Device Evaluation, National Medical Products Administration, Beijing 100081, China; 3 School of Life Sciences, Beijing University of Chinese Medicine, Beijing 100029, China

**Keywords:** Alzheimer’s, endophytic fungi, secondary metabolites, natural products, bioactivities

## Abstract

Alzheimer’s is the most common cause of dementia worldwide and seriously affects patients’ daily tasks. Plant endophytic fungi are known for providing novel and unique secondary metabolites with diverse activities. This review focuses primarily on the published research regarding anti-Alzheimer’s natural products derived from endophytic fungi between 2002 and 2022. Following a thorough review of the literature, 468 compounds with anti-Alzheimer’s-related activities are reviewed and classified based on their structural skeletons, primarily including alkaloids, peptides, polyketides, terpenoids, and sterides. The classification, occurrences, and bioactivities of these natural products from endophytic fungi are summarized in detail. Our results provide a reference on endophytic fungi natural products that may assist in the development of new anti-Alzheimer’s compounds.

## 1. Introduction

Alzheimer’s disease (AD) is a progressive neurodegenerative disorder and the most common cause of dementia worldwide that affects memory, thinking, and behavior and even interferes with daily tasks. The abnormal accumulation of beta-amyloid and phosphorylated tau proteins and nerve cell degeneration are deemed to play key roles in Alzheimer’s disease [1,2]. According to the latest WHO report, the number of people suffering from dementia worldwide in 2010 was about 35.6 million, while the figure could be triple this by 2050 [1]. Age is the biggest risk factor for Alzheimer’s dementia, which dramatically increases the incidence and death rate of Alzheimer’s dementia and contributes to a heavy burden on families and society. The incidence of dementia was 5.0–13.1% for people over 65 years old, while this number increased to 33.2% as the age rose to over 85 years of age, and the death rate increased by 33–51% for people over 65 years of age and by 78% for people aged 80 and older [3]. Only a few therapeutic agents have been made clinically available for this disease, such as memantine, donepezil, rivastigmine, tacine, galantamine, and aducanumab [4,5,6]. These drugs can relieve AD-related symptoms for mild cognitive impairment, but are incapable of preventing disease progression to obtain ideal treatment effects [7]. Thus, it is critical to develop new treatments for AD to prevent and delay the progression of the disease, improve cognition, and reduce the behavioral disorders of patients with AD.

Endophytic fungi were first identified in plants in 1809 [8]. They are microorganisms that reside in the tissues of healthy plants for part of or all of their life cycle without causing apparent infection in the host plant. Some endophytes provide new bioactive compounds with unique structures containing alkaloids, phenols, lactones, quinones, terpenoids, steroids, and other compounds. These isolated metabolites display antibiotic, antioxidant, anti-inflammatory, antiviral, and anti-Alzheimer’s properties, among others [9,10,11,12]. 

In this review, a comprehensive survey of approximately 468 compounds with anti-Alzheimer’s-related activities derived from endophytic fungi from 2002 to 2022 is performed. These compounds are classified by their structure skeleton and mainly include alkaloids, peptides, polyketides, terpenoids, and sterides. The most investigated activities of these metabolites are the inhibition of acetylcholinesterase (AChE), butyrylcholinesterase (BChE), neuroprotection, *β*-site amyloid precursor protein-cleaving enzyme 1 (BACE1) inhibition, and their antioxidant activities. So far, the secondary metabolites of plant endophytic fungi with anti-Alzheimer’s activities have not been summarized. This review mainly focuses on the classification, occurrences, and bioactivites of the secondary metabolites derived from endophytic fungi.

## 2. Bioactive Compounds from Plant Endophytic Fungi

### 2.1. Alkaloids

#### 2.1.1. Cytochalasans

**Figure 1:**
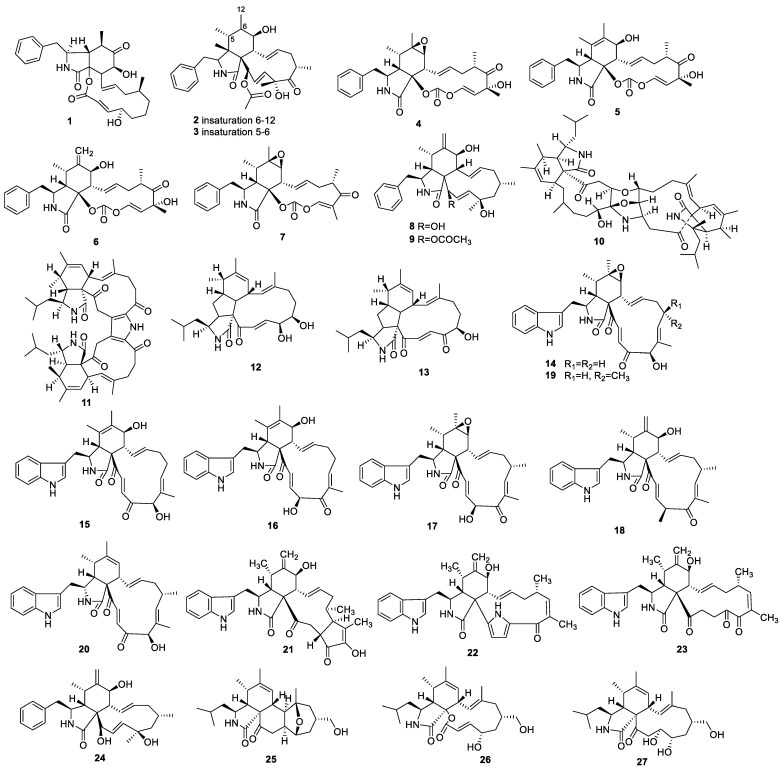
Chemical structures of cytochalasans (**1**–**27**).

The chemical study of endophyte *Xylaria* sp. collected from the leaves of *Casearia sylvestris* showed cytochalasins B–D (**1**–**3**) (Figure 1). Compounds **1** and **2** showed strong anti-AChE activities at 60 µg [13]. Research on *Aspergillus terreus* obtained from the stems of *Artemisia annua* afforded the four known cytochalasans cytochalasin E (**4**), 5,6-dehydro-7-hydroxy cytochalasin E (**5**), Δ^6,12^-isomer of 5 (**6**), and rosellichalasin (**7**). Compounds **4**–**7** showed weak anti-AChE activities with IC_50_ from 110.9 to 176.0 μM [14]. Cytochalasins J (**8**) and H (**9**) were identified from endophyte *Phomopsis* sp., which was isolated from *Senna spectabilis* (Fabaceae). Compound **9** demonstrated AChE inhibition in vitro at a minimum quantity of 25.0 µg [15]. 

Two heterocycle-fused cytochalasan homodimers, bisaspochalasins D (**10**) and E (**11**), along with aspochalasins D (**12**) and B (**13**), were identified from an endophytic *Aspergillus flavipes* associated with the stems of *Hevea brasiliensis*. Among them, compound **10** alone exhibited neurotrophic activities that could accelerate neurite growth with a differentiation rate of 12.52% for rat pheochromocytoma cells (PC12) at 10 μM [16]. 

Seven compounds containing chaetoglobosins A (**14**), B (**15**), E (**16**), F (**17**), and F_ex_ (**18**) as well as penochalasins F (**19**) and G (**20**) were separated from *Chaetomium globosum*, an endophytic fungus associated with the seeds of *Panax notoginseng*. Compound **14** showed negligible anti-AChE activity with an inhibition ratio of less than 10% at 50 μM. None of them showed 2,2-diphenyl-1-picrylhydrazyl (DPPH) free radical-scavenging activity with half effective concentration (EC_50_) greater than 100 ug/mL [17].

A total of six 10-indolyl-cytochalasans (**16**–**18**), cytoglobosin A (**21**), penocha-lasin C (**22**), and isochaetoglobosin D (**23**), were collected from *Chaetomiun globosum* WQ in *Imperata cylindrical*, and **9** and 18-methoxycytochalasin J (**24**) were identified from *Phomopsis* sp. IFB-E060 in *Vatica mangachapoi*. With the exception of **22**, these compounds showed scavenging DPPH activity with an EC_50_ between 0.002 ± 0.001 and 1.068 ± 0.350 mM, scavenging ABTS activity with an EC_50_ between 0.002 ± 0.001 and 1.052 ± 0.357 mM, strong inhibiting activity of hydrogen peroxide (H_2_O_2_)-mediated PC12 cell damage with an EC_50_ between 0.003 ± 0.0003 and 0.240 ± 0.236 µM, and inhibiting N-methyl-4-phenylpyridinium iodide (MPP^+^) induced PC12 cell damage activity with an EC_50_ between 0.009 ± 0.007 and 6.100 ± 0.007 µM [18].

Chemical research on mangrove endophyte *Westerdykella nigra* collected from the roots of *Avicennia marina* (Forssk.) Vierh. resulted in the isolation of westalsan (**25**), phomacin B (**26**), and 19-hydroxy-19,20-dihydrophomacin C (**27**), which showed apparent AChE inhibition with IC_50_s of 0.056 ± 0.003, 0.088 ± 0.005, and 0.140 ± 0.007 μM, respectively [19].

#### 2.1.2. Diketopiperazine Derivatives

**Figure 2:**
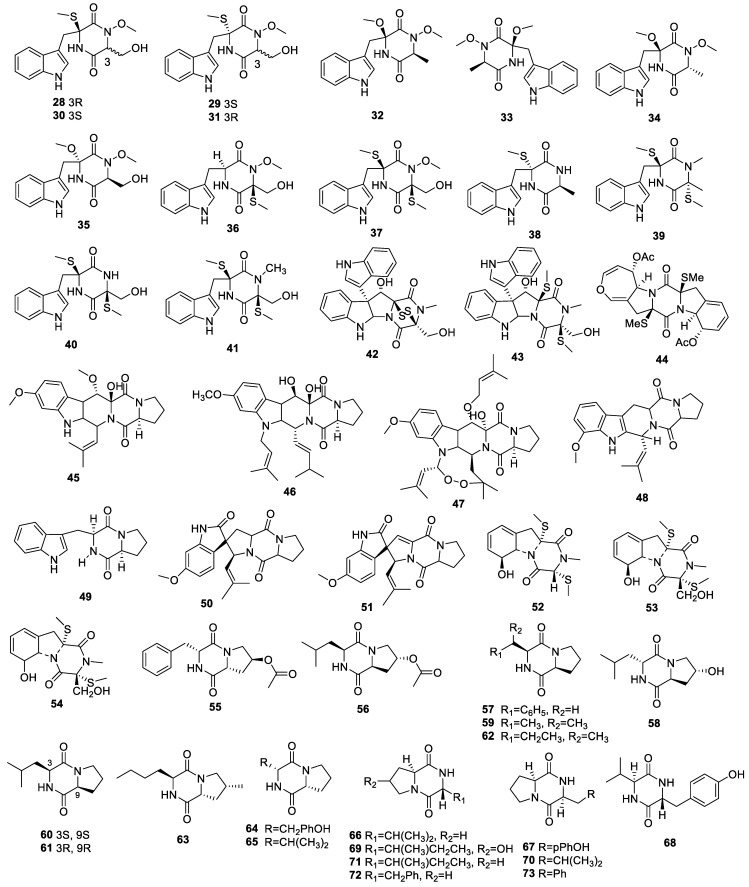
Chemical structures of diketopiperazine derivatives (**28**–**73**).

Detailed chemical research on an endophytic fungus, *Acrostalagmus luteoalbus* TK-43, collected from marine green alga *Codium fragile,* led to the identification of three pairs of indolediketopiperazine enantiomers (acrozine A (**28/29**), acrozine B (**30/31**), and acrozine C (**32/33**)), four new congeners (acrozines D–G, **34**–**37**), and six known analogs (pseudellone D (**38**), lasiodipine E (**39**), chetoseminudin C (**40**), chetoseminudin B (**41**), T988 C (**42**), and T988D (**43**)) (Figure 2). Compounds **28**–**37** possessed an unusual methoxy site in N-2, which was scarcely reported in indolediketopiperazine alkaloids. The evaluation of compounds **28**–**37** for anti-AChE activity revealed that compound **28** displayed better inhibition with an IC_50_ of 2.3 µM than did **29** (IC_50_ = 13.8 µM). Compounds **30**–**33** demonstrated moderate and weak AChE inhibitory activities with IC_50_ values in the range of 49.2 to 160.6 µM [20]. The IC_50_ for AChE inhibition by compound **36** was 8.4 μM. Other compounds showed weak activities at 32 μM [21].

Acetylapoaranotin (**44**) was identified from the liquid fermentation of *Aspergillus terreus* associated with the stems of *Artemisia annua*. The IC_50_ of compound **44** for anti-AChE activity was 127.4 μM [14]. 

Three known alkaloids, cyclotryprostatin B (**45**), fumitremorgin B (**46**), and fumitremorgin A (**47**), were isolated from the endophyte *Neosartorya fischeri* JS0553 of *Glehnia littoralis*. None of these alkaloids showed obvious neuroprotection against glutamate-mediated HT22 cell injury at 20 μM [22]. 

Fumitremorgin C (**48**), brevianamide F (**49**), spirotryprostatin A (**50**), 6-methoxyspirotryprostatin B (**51**), 3-dehydroxymethylbisdethio-3,10a-bis(methylthio)gliotoxin (**52**), bisdethiobis(methylthio)gliotoxin (**53**), and didehydrobisdethiobis(methylthio)gliotoxin (**54**) were collected from endophyte *Talaromyces* sp. LGT-2 associated with *Tripterygium wilfordi*. Compound **53** showed weaker anti-AChE activity [23].

A chemical study of *Nigrospora camelliae-sinensis* S30 collected from mangrove *Lumnitzera littorea* afforded two new 2,5-diketopiperazine derivatives, nigrosporaamides A and B (**55**, **56**), and seven known analogs (**57**–**63**): cyclo-(L-Pro-L-Phe) (**57**), cyclo[L-(4-hydroxyprolinyl)-L-Leu] (**58**), cyclo-(L-Val-L-Pro) (**59**), cyclo-(L-Leu-L-Pro) (**60**), cyclo-(R-Leu-R-Pro) (**61**), cyclo-(L-Ile-L-Pro) (**62**), and cyclo-(4-methyl-R-Pro-S-Nva) (**63**). None showed significant neuroprotection against H_2_O_2_-mediated cytotoxicity for HT22 cells at 100 μM [24]. In addition, compound **59** was also discovered in potato dextrose broth fermentation cultures of *Penicillium* sp.1, an endophytic fungi living in the leaves of *Alibertia macrophylla* (Rubiaceae), which exhibited potent AChE inhibition with a detection limit of 10.0 μg [25].

Diketopiperazines cyclo-(S-Pro-S-Tyr) (**64**) and cyclo-(S-Pro-S-Val) (**65**) were isolated from *Colletotrichum gloeosporioides* [26]. Cyclo(D)-Pro-(L)-Val (**66**), cyclo(D)-Pro-(D)-Tyr (**67**), cyclo(D)-Val-(D)-Tyr (**68**), cyclo(D)-Hyp-(L)-Ile (**69**), cyclo(D)-Pro-(D)-Leu (**70**), cyclo(D)-Pro-(L)-Ile (**71**), cyclo(D)-Pro-(L)-Phe (**72**), and cyclo(D)-Pro-(D)-Phe (**73**) were isolated from *Colletotrichum crassipes* [13]. Among them, compounds **64** and **65** exhibited moderate AChE inhibitory activities at 200 μg [26].

#### 2.1.3. Indole Alkaloids

**Figure 3:**
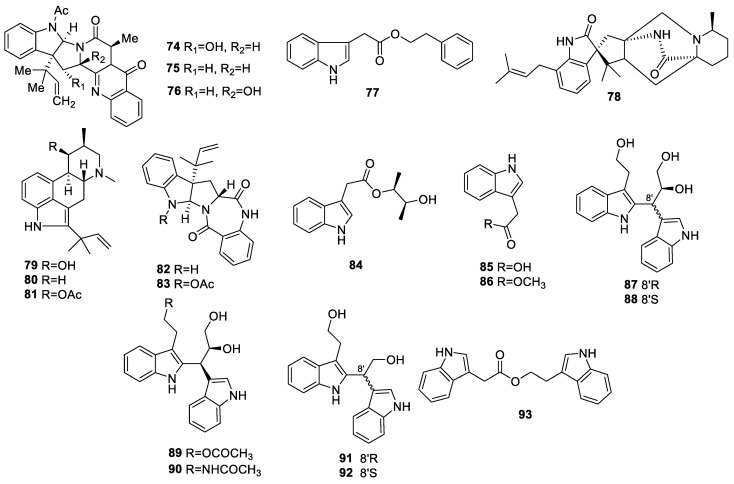
Chemical structures of indole alkaloids (**74**–**93**).

One new alkaloid, 16α-hydroxy-5N-acetylardeemin (**74**), together with two known compounds, 5N-acetylardeemin (**75**) and 15b-β-hydroxy-5N-acetylardeemin (**76**) (Figure 3), were identified from the liquid fermentation of the endophyte *Aspergillus terreus* of *Artemisia annua*. Compounds **74**–**76** displayed anti-AChE activities with IC_50_ values of 58.3, 149.4, and 116.9 µM, respectively [14]. 

A chemical study of the endophytic fungus *Colletotrichum gloeosporioides* collected from the leaves of *Michelia champaca* led to the isolation of a new compound, 2-phenylethyl 1H-indol-3-yl-acetate (**77**), which exhibited moderate AChE inhibitory activity at 200 μg during a bioautography analysis [26].

A new macfortine alkaloid, chrysogenamide A (**78**), was identified from *Penicillium chrysogenum* No. 005, an endophyte from the root of *Cistanche deserticola*. Compound **78** showed no scavenging DPPH free radical activity at 100 µM, while it exhibited the inhibition of H_2_O_2_-mediated SH-SY5Y cell death by enhancing cell viability by 59.6% at 1 × 10^−4^ μM, suggesting that **78** exhibited a protective effect on neurocytes via oxidative stress-mediated cell death in SH-SY5Y cells rather than through antioxidant activity [27].

An investigation of the endophytic fungus *Aspergillus fumigatus* of *Cynodon dactylon* revealed two new alkaloids, 9-deacetylfumigaclavine C (**79**) and 9-deacetoxyfumigaclavine C (**80**), as well as the known compound fumigaclavine C (**81**). These isolates were practically inactive to induce the neurie outgrowth of PC12 [28].

Two known alkaloids, aszonalenin (**82**) and acetylaszonalenin (**83**), were identified from *Neosartorya fischeri* JS0553, an endophyte of *Glehnia littorali*. Neither showed obvious neuroprotection against glutamate-induced HT22 cell damage [22].

A new indole alkaloid, alternatine A (**84**), and two known indole alkaloids, 1*H*-indole-3-carboxylic acid (**85**) and indole-3-methylethanoate (**86**), were identified from *Alternaria alternate* collected from the leaves of *Psidium littorale* Raddi. The cell viabilities of **86** were prominently increased by 75.6 ± 4.2% and 84.8 ± 6.5% at 40 and 80 μM, respectively [29]. Compound **85** was also identified in *Epicoccum nigrum* and *Penicillium brefeldianum* F4a collected from the fresh leaves of *Entada abyssinica* Steud. ex A. Rich. Fabaceae and the roots of *Houttuynia cordata*, respectively. This compound exhibited weak scavenging activity with IC_50_ = 88.97 µg/mL in the DPPH assay and EC_50_ = 21.48 ± 0.88 µM in the ABTS assay [30,31]. 

Seven dimeric tryptophol-related alkaloids, colletotryptins A–D (**87**–**90**), E (**91**/**92**), and F (**93**), were separated from the solid fermentation of *Colletotrichum* sp. SC1355, an endophytic fungus collected from the healthy leaves of *Cleistocalyx operculatus*. Compounds **87**–**93** did not show AChE inhibitory activity [32].

#### 2.1.4. Other Alkaloids

**Figure 4:**
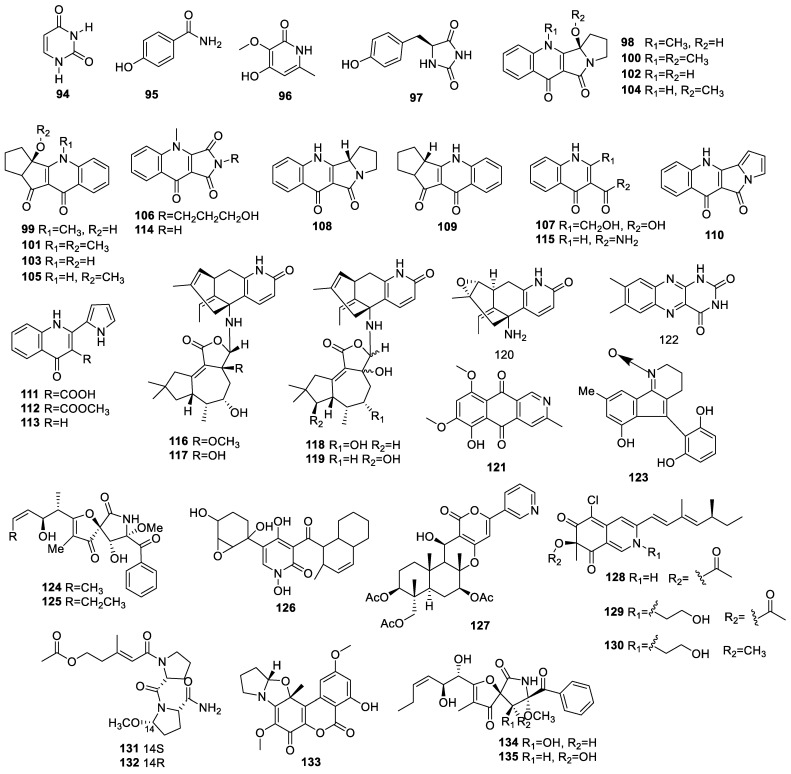
Chemical structures of other alkaloids (**94**–**135**).

The chemical investigation of endophyte *Colletotrichum gloeosporioides* identified in the leaves of *Michelia champaca* revealed two known compounds, uracil (**94**) and 4-hydroxy-benzamide (**95**) (Figure 4), which exhibited moderate AChE inhibitory activities at 200 μg [26].

One new metabolite, α-pyridone derivative 3-hydroxy-2-methoxy-5-methylpyridin-2(1H)-one (**96**), was isolated from *Botryosphaeria dothidea* KJ-1, an endophytic fungus from the stems of *Melia azedarach* L. This compound showed low DPPH scavenging activity with a rate of 22.5% at 50 μM [33].

One known compound, 5-(40-Hydroxybenzyl) hydantoin (**97**), identified from *Nigrospora camelliae-sinensis* S30 associated with mangrove *Lumnitzera littorea*, was not found to exhibit obvious neuroprotective activity against H_2_O_2_-mediated cytotoxicity for HT22 cells [24].

Four new racemic mixtures of 4-quinolone alkaloids, (±)-oxypenicinolines A (**98**/**99**); B (**100**/**101**); C (**102**/**103**); and D (**104/105**), and two congeners, penicinolines F (**106**) and G (**107**), as well as seven known related compounds, 1,2,3,11b-tetrahydroquinolactacide (**108/109**); quinolactacide (**110**); penicinoline (**111**); methyl-penicinoline (**112**); penicinoline E (**113**); quinolonimide (**114**); and 4-oxo-1 and 4-dihydroquinoline-3-carboxamide (**115**), were collected from *Penicillium steckii* SCSIO 41025 (Trichocomaceae), a mangrove-derived endophyte of *Avicennia marina* (Forsk.) Vierh (Trichocomaceae). Only compounds **111** and **113** showed mild AChE inhibition with IC_50_s of 87.3 and 68.5 μM, respectively [34].

Endophyte *Ceriporia lacerate* HS-ZJUT-C13A identified in the medicinal plant *Huperzia serrata* was chosen for transforming hupA in a liquid potato–dextrose medium. Five unusual alkaloids, huptremules A–D (**116**–**119**) and 8α,15α-epoxyhuperzine A (**120**), were obtained. Among them, **116**–**119** characterized irregular sesquiterpenoid–alkaloid structural hybrids, which combined the features of fungal metabolites and the substrate of hupA. These isolates displayed significant AChE inhibition with IC_50_ within a range of 0.06 to 12.11 μM (positive control hupA with an IC_50_ of 0.54 μM) [35].

Chemical research on *Aspergillus terreus* (No. GX7-3B), a mangrove endophytic fungus from a branch of *Bruguiera gymnoihiza* (Linn.) Savigny, resulted in the isolation of 8-O-methylbostrycoidin (**121**), which showed prominent AChE inhibition with IC_50_ at 6.71 μM [36].

A study on the endophytic fungus *Fusarium* sp. HP-2 identified the compound lumichrome (**122**), which did not exhibit AChE inhibition at 50 μM [37].

An investigation of *Phomopsis* sp. xy21 related to leaves of the Thai *Xylocarpus granatum* isolated phomopsol A (**123**) with a matchless 3,4-dihydro-2H-indeno [1,2-b]pyridine 1-oxide group. The cell activities of **123** were 76% at 40.0 μM, which showed neuroprotection against corticosterone-mediated PC12 cell injury with a concentration-dependent effect within the scope of 5.0–40.0 μM [38].

Two known compounds, 14-norpseurotin (**124**) and pseurotin A (**125**), were identified from *Aspergillus fumigatus* related to a healthy stem of *Cynodon dactyl*. Compound **124** had stronger activity than did **125** in promoting neurite outgrowth at 10.0 µM for PC12 [28].

Chemical research on *Neosartorya fischeri* JS0553 associated with *Glehnia littoralis* produced two known alkaloids: fischerin (**126**) and pyripyropene A (**127**). The investigation of the mechanisms for glutamate-induced HT22 cell injury revealed that **126** could inhibit Ca^2+^ influx, ROS, and the phosphorylation of JNK, ERK, and p38 to exert conspicuous neuroprotection [22].

Three new alkaloids, penazaphilone E (**128**), isochromophilone VI (**129**), and peniazaphilone D (**130**), were identified from *Penicillium* sp. JVF17 related to *Vitex rotundifolia*. Compounds **128**–**130** have been proven to possess almost 100% neuroprotection at 25 μM. The mechanism of **128** regarding glutamate-mediated HT22 cell death involved restraining MAPKs phosphorylation and reducing the Bax/Bcl-2 ratio [39].

An investigation of *Cochliobolus lunatus* SCSIO41401 led to the isolation of the lipopeptide epimers sinulariapeptides A (**131**) and B (**132**), which displayed obvious AChE inhibition with IC_50_s of 1.8 ± 0.12 and 1.3 ± 0.11 μM, respectively [40].

Research on the endophytic fungus *Rhizopycnis vagum* Nitaf22 revealed a novel alkaloid, rhizovagine A (**133**), which has an unprecedented 5/5/6/6/6 integrated pentacyclic skeleton. This compound was found to exhibit AChE inhibition with an IC_50_ of 43.1 μM [41].

The study of *Talaromyces* sp. LGT-2 associated with *Tripterygium wilfordi* resulted in the identification of pseurotin A1 (**134**) and pseurotin A2 (**135**), which showed weaker anti-AChE activities [23].

### 2.2. Peptides

**Figure 5:**
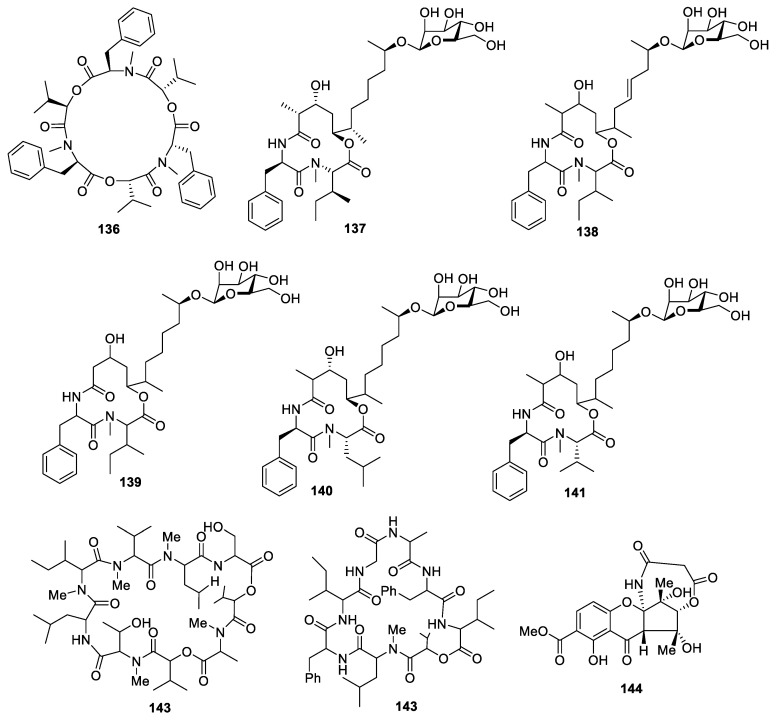
Chemical structures of peptides (**136**–**144**).

Beauvericin (**136**) (Figure 5) was collected from *Aspergillus terreus* (No. GX7-3B) from a branch of *Bruguiera gymnoihiza* (Linn.) Savigny. The IC_50_ of this compound for AChE inhibition was 3.09 μM [36].

Colletotrichamides A−E (**137**–**141**) were identified from *Colletotrichum gloeosporioides* JS419, a fungus collected from *Suaeda japonica* Makino. Colletotrichamide C (**139**) displayed potent neuroprotection with 100% cell activity at 100 μM against glutamate-induced HT22 cell death [42].

The study of *Bipolaris sorokiniana* LK12 led to the isolation of BZR-cotoxin I (**142**) and BZR-cotoxin IV (**143**), which possessed mild anti-AChE, lipid peroxidation, and urease activities [43].

A chemical study of *Cryptosporiopsis* sp. identified cryptosporioptide (**144**), which possessed significant lipoxygenase inhibition with an IC_50_ of 49.15 ± 0.17 µM [44].

### 2.3. Polyketides

#### 2.3.1. Pyranones and Pyranyl Derivatives

##### Simple Pyranones

**Figure 6:**
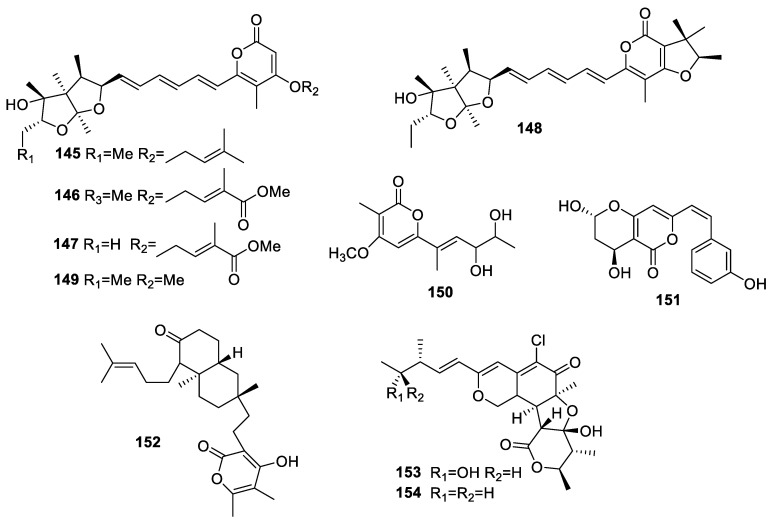
Chemical structures of simple pyranones (**145**–**154**).

Four new prenylated asteltoxin analogs, avertoxins A–D (**145**–**148**), along with the known mycotoxin asteltoxin (**149**) (Figure 6) were obtained from *Aspergillus* *versicolor* Y10 associated with the leaves of *Huperzia serrata*. The IC_50_ of avertoxin B (**146**) for AChE inhibition was 14.9 μM [45].

A study on *Xylaria* sp. HNWSW-2 collected from the stem of *Xylocarpus granatum* led to the isolation of astropyrone (**150**), which diaplayed weak anti-AChE activity with an inhibition rate of 10.4% at 50 µg/mL [46].

The investigation of *Bipolaris sorokiniana* LK12 identified in *Rhazya stricta* revealed the isolation of bipolarisenol (**151**), which showed obvious AChE inhibition with an IC_50_ of 67.23 ± 5.12 µg/mL and also displayed mild lipid peroxidation inhibition with an IC_50_ of 168.91 ± 4.23 µg/mL [47].

Pycnophorin (**152**) was collected from *Botryosphaeria dothidea* KJ-1, which presented as a weak DPPH scavenger with a scavenging rate of 22.5% at 50 μM [34].

A chemical study of *Chaetomium globosum* associated with the seeds of *Panax notoginseng* led to the isolation of chaetomugilins A (**153**) and D (**154**). Neither showed antioxidant activities with an EC_50_ greater than 100 μg/mL in DPPH free radical scavenging [17].

##### Benzopyrones

**Figure 7:**
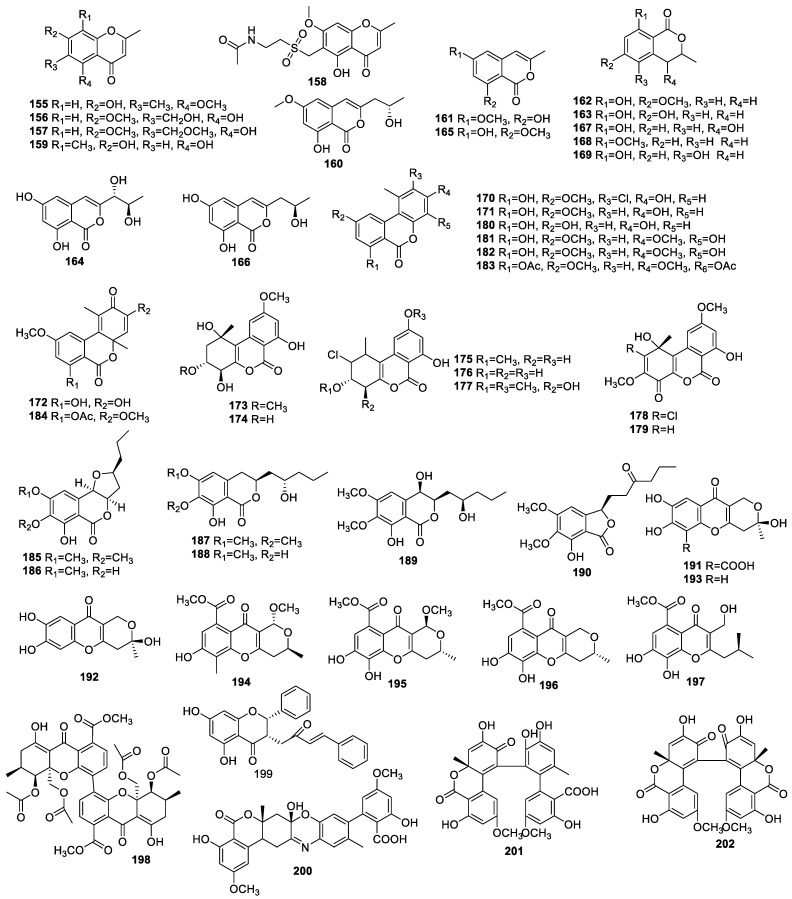
Chemical structures of benzopyrones (**155**–**202**).

Chromone derivatives hydroxylchromone (**155**) (Figure 7); 6-hydroxymethyleugenin (**156**); 6-methoxymethyleugenin (**157**); chaetoquadrin D (**158**); isoeugenitol (**159**) and isocoumarin congeners diaporthin (**160**); 8-hydroxy-6-methoxy-3-methylisocoumarin (**161**); and 6-methoxymellein (**162**) were isolated from *Xylomelasma* sp. Samif07 related to *Salvia miltiorrhiza* Bunge. Compound **160** alone displayed powerful antioxidant activity with an EC_50_ of 15.1 µg/mL in hydroxyl radical scavenging [48].

I-6-hydroxymellein (**163**), 6,8-dihydroxy-3-(10R, 20R-dihydroxypropyl)-isocoumarin (**164**), 6-hydroxy-8-methoxy-3-methylisocoumarin (**165**), and de-O-methyldiaporthin (**166**) were collected from *Phaeosphaeria* sp. LF5 associated with the leaves of *Huperzia serrata*. The IC_50_ value of compound **166** for AChE inhibition was 21.18 µM. Other compounds showed inactivity at 100 µM [49].

4-Hydroxymellein (**167**), 8-methoxymellein (**168**), and 5-hydroxymellein (**169**) were isolated from *Penicillium* sp.2 collected from the leaves of *Alibertia macrophylla* (Rubiaceae). This was the first time compounds **168** and **169** had been isolated from the genus *Penicillium*. These compounds demonstrated moderate to weak AChE inhibitory activities [25].

α-Pyrone derivatives (**167**, **170**–**181**) containing 4-hydroxymellein (**167**), palmariol B (**170**), alternariol 9-methyl ether (**171**), botrallin (**172**), hyalodendriols A–C (**173**–**175**), rhizopycnin D (**176**), penicilliumolide D (**177**), TMC-264 (**178**), penicilliumolide B (**179**), alternariol (**180**), and graphislactone A (**181**) were obtained from *Hyalodendriella* sp. Ponipodef 12, an endophyte from the hybrid ‘N’va’ of *Populus deltoides* Marsh × *P. nigra* L. L. Compounds **170**–**172**, **174**, **178**, and **179** exhibited moderate to weak activities for AChE inhibition with IC_50_ values within the scope of 21.1 to 135.52 μg/mL. Other compounds were inactive with an IC_50_ beyond 200 µM for anti-AChE activities [50,51].

Four known compounds, including graphislactone A (**182**), graphislactone A diacetate (**183**), botrallin (**172**), and botrallin diacetate (**184**), were isolated and identified from *Microsphaeropsis olivacea* obtained from *Pilgerodendron uviferum* (D. Don) Florin (“Cipres de las Guaitecas”). Compounds **182**, **183**, **172**, and **184** showed strong to moderate AChE inhibitory activities with IC_50_s of 8.1, 88, 6.1, and 27 µg/mL, respectively [52].

Five isocoumarins, monocerin (**185**); monocerin demethylated derivative (**186**); fusarentin 6,7-dimethyl ether (**187**); fusarentin 6-methyl ether (**188**); fusarentin derivative (**189**); and phthalide (**190**) were collected from the *Colletotrichum* sp. CRI535-02 of *Piperornatum*. The IC_50_s of compounds **186** and **188** were 23.4 and 16.4 µM for DPPH inhibition and 52.6 and 4.3 µM for superoxide anion radical inhibition, respectively. Isocoumarins **185**–**187** showed excellent ORAC antioxidation with 10.8–14.4 ORAC units, and **190** displayed antioxidation with 2.4 units [53].

Penialidin A (**191**), penialidin F (**192**), and myxotrichin C (**193**) were identified from *Penicillium brefeldianum* F4a associated with the roots of *H. cordata*. Compounds **192** and **193** could scavenge DPPH with EC_50_s of 28.42 ± 3.16 and 30.07 ± 2.83 µM, respectively. Compounds **191**–**193** had the strongest scavenging ABTS^+^ activity with EC_50_s of 14.54 ± 0.46, 7.61 ± 0.46, and 14.96 ± 2.57 µM, respectively [31].

A detailed chemical study of *Phomopsis* sp. 33#., an endophytic fungus from *Rhizophora stylosa*, led to the discovery of four new compounds, phomopsichins A–D (**194**–**197**), and the known compound phomoxanthone A (**198**). Compounds **194**–**198** showed weak inhibitory activities against AChE with an inhibitory rate from 2.7% to 38.4% for a concentration of 250 µM and displayed weak scavenging DPPH activity with an inhibitory rate from 17.0% to 52% at 1 mM [54].

A new compound, (2R,3S)-pinobanksin-3-cinnamate (**199**), isolated from the endophytic fungus *Penicillium* sp. FJ-1 of *Acanthus ilicifolius* Linn, exhibited a potent neuroprotective effect on corticosterone-damaged PC12 cells [55].

Three novel aromatic polyketide dimers, bialternacin A (**200**), bialternacin E (**201**), and bialternacin F (**202**), featured as racemic mixtures, were identified from a plant endophytic *Alternaria* sp. associated with stem of *Maianthemum bifolium*. Compound **192** alone exhibited AChE inhibition with an IC_50_ of 15.5 μM [56].

##### Pyranyl Derivatives

**Figure 8:**
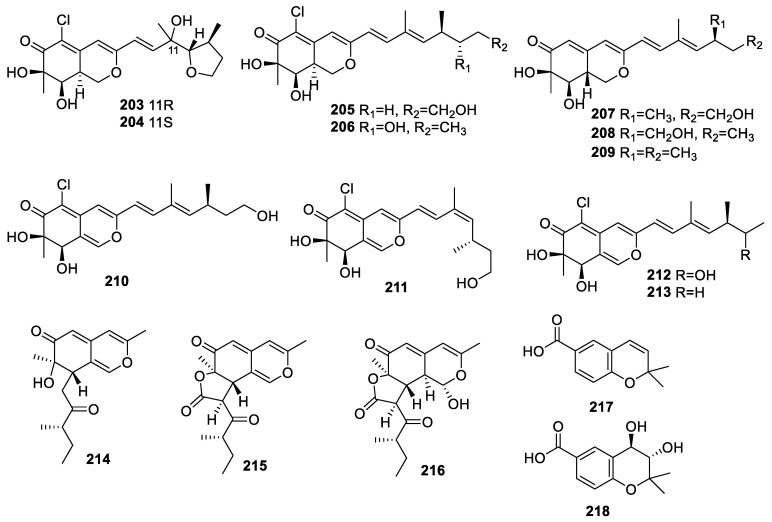
Chemical structures of pyranyl derivatives (**203**–**218**).

A chemical study of *Penicillium* sp. JVF17 associated with *Vitex rotundifolia* led to the isolation of nine azaphilone-type polyketides, peniaphilones A–I (**203**–**208**, **210**–**212**), together with dechloroisochromophilone III (**209**) and isochromophilone V (**213**) (Figure 8). Compounds **205, 208**, **209**, and **213** showed neuroprotective effects against glutamate-induced HT22 cell injury within the scope of 25 μM and 100 μM [39].

Three new azaphilones, chermesinones A–C (**214**–**216**), were collected from *Penicillium chermesinum* (ZH4-E2) associated with the stem of *Kandelia candel*. None exhibited the inhibition of AChE (IC_50_ > 100 μM) [57].

The chemical investigation of *Saccharicola* sp. isolated from *Eugenia jambolana* resulted in the identification of two compounds: 2,2-dimethyl-2H-chromene-6-carboxylic acid (**217**) and *trans*-3,4-dihydro-3,4-dihydroxy-anofinic acid (**218**). Compound **218** displayed huAChE-ICER and eeAChE-ICER inhibitory activities with IC_50_s of 0.037 ± 0.01 and 0.026 ± 0.005 mg/mL, respectively [58].

#### 2.3.2. Quinones

**Figure 9:**
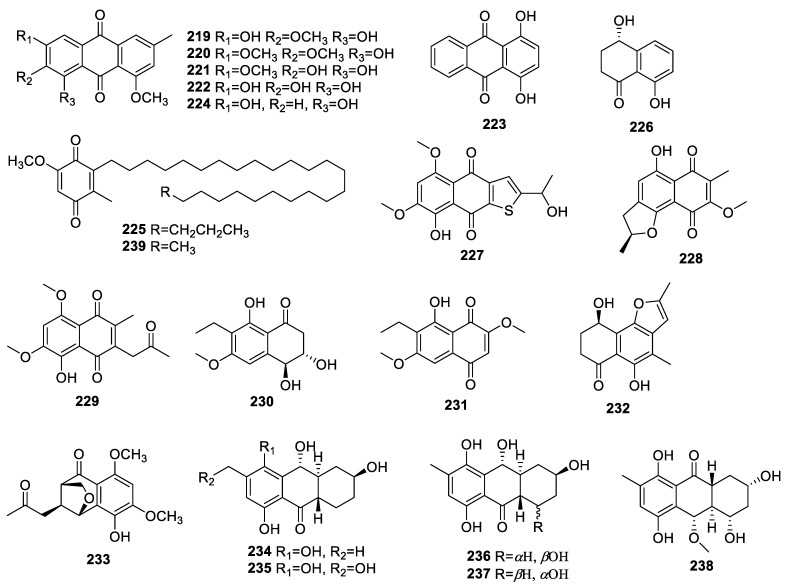
Chemical structures of quinones (**219**–**239**).

The chemical investigation of *Colletotrichum* sp. JS-0367 associated with the leaves of *Morus alba* (mulberry) led to the identification of the new compound 1,3-dihydroxy-2,8-dimethoxy-6-methylanthraquinone (**219**) and the three known compounds 1-hydroxy-2,3,8-trimethoxy-6-methylanthraquinone (**220**), 1,2-dihydroxy-3,8-dimethoxy-6-methylanthraquinone (**221**), and evariquinone (**222**) (Figure 9). Compound **222** inhibited intracellular ROS aggregation, Ca^2+^ influx, MAPKs phosphorylation, and apoptotic cell death to exert potent protection against glutamate-mediated HT22 cell death [59].

Quinizarin (**223**) identified from *Epicoccum nigrum,* an endophyte from the fresh leaves of *Entada abyssinica* Steud. ex A. Rich., Fabaceae, exhibited significant ABTS and DPPH scavenging activities with IC_50_s of 10.86 and 11.36 µg/mL, respectively [30].

A chemical study of the *Chaetomium* sp. YMF432 of *Huperzia serrata* (Thunb. ex Murray) Trev led to the discovery of known compounds 1-omethylemodin (**224**), 5-methoxy-2-methyl-3-tricosyl-1,4-benzoquinone (**225**), and isosclerone (**226**), which were identified in this fungus for the first time. Compounds **224** and **225** displayed mild AChE inhibition with IC_50_s of 37.7 ± 1.5 and 37.0 ± 2.9 μM, respectively, while compound **226** was inactive for anti-AChE activity with an inhibition rate of less than 10% at 100 μg/mL [60]. In addition, isosclerone (**226**) was also identified from *Alternaria alternate* collected from the leaves of *Psidium littorale* Raddi., which showed neuroprotective activities for glutamate-injured PC12 cells by significantly improving cell viabilities with values ranging from 65.9 ± 3.9% to 74.6 ± 4.0% after treatment with the compound at 20, 40, and 80 μM [29].

Research on *Aspergillus terreus* (No. GX7-3B) related to a branch of *Bruguiera gymnoihiza* (Linn.) revealed the identification of an unusual thiophene, 8-hydroxy-2-[1-hydroxyethyl]-5,7-dimethoxynaphtho[2,3-*b*] thiophene-4,9-dione (**227**), as well as anhydrojavanicin (**228**), 8-O-methyljavanicin (**229**), botryosphaerone D (**230**), and 6-ethyl-5-hydroxy-3,7-dimethoxynaphthoquinone (**231**). The IC_50_ of **228** for anti-AChE activity was 2.01 μM [36].

An investigation into *Fusarium* sp. HP-2 from “Qi-Nan” agarwood found two new naphthalenone analogs: 3-demethoxyl-fusarnaphthoquinone B (**232**) and (2*S*,3*S*,4*S*)-8-dehydroxy-8-methoxyl-dihydronaphthalenone (**233**). The inhibition ratio of **233** against AChE was 11.9% at 50 μM [37].

A detailed chemical study on endophyte *Talaromyces islandicus* EN-501 associated with red alga *Laurencia okamurai* led to the isolation of 8-hydroxyconiothyrinone B (**234**), 8,11-dihy-droxyconiothyrinone B (**235**), 4R,8-dihydroxyconiothyrinone B (**236**), 4S,8-dihydroxyconiothyrinone B (**237**), and 4S,8-dihydroxy-10-O-methyldendryol E (**238**). Compounds **234**−**238** exhibited antioxidant activities with IC_50_ = 12, 31, 42, 52, and 30 μM against DPPH and IC_50_ = 8.3, 19, 34, 31, and 24 μM against ABTS, respectively [61].

5-Methoxy-2-methyl-3-pentacosylcyclohexa-2,5-diene-1,4-dione (**239**) identified from the *Colletotrichum* sp. F168 of the plant *Huperzia serrata* Trev displayed negligible AChE inhibition at 10.9% at 100 µg/mL [62].

#### 2.3.3. Other Polyketides

**Figure 10:**
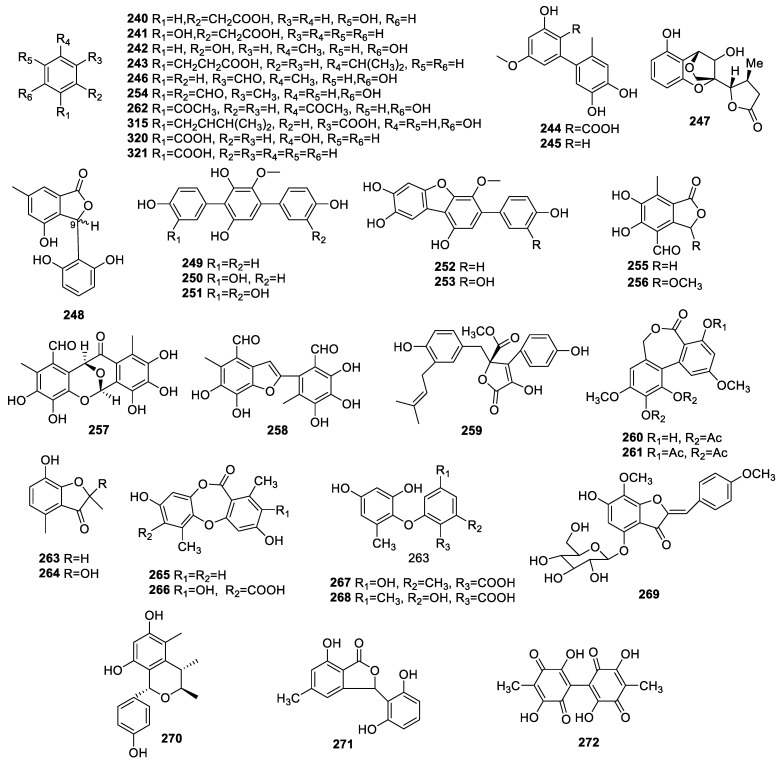
Chemical structures of other polyketides (**240**–**272**, **315**, **320**, and **321**).

Compounds 2(4-hydroxyphenyl)acetic acid (**240**) and 2(2-hydroxyphenyl)acetic acid (**241**) (Figure 10) were identified from the endophyte *Colletotrichum gloeosporioides*. These compounds exhibited mild anti-AChE activity at 200 μg via bioautography [26].

Orcinol (**242**) was obtained from *Penicillium* sp.1, an endophytic fungus from the leaves of *Alibertia macrophylla* (Rubiaceae). It exhibited moderate AChE inhibition [25].

One bioactive compound, sorokiniol (**243**), was isolated from fungal endophyte *Bipolaris sorokiniana* LK12. It exhibited significant AChE inhibition with an EC_50_ of 3.402 ± 0.08 μg/mL [43].

The chemical assay for endophyte *Botryosphaeria dothidea* KJ-1 led to the qualification of altenusin (**244**) and 5′-methoxy-6-methylbiphenyl-3,4,3′-triol (**245**), which displayed obvious DPPH scavenger activities with an IC_50_ of 17.6 ± 0.23 and 18.7 ± 0.18 μM, respectively [33].

Parahydroxybenzaldehyde (**246**) collected from *Epicoccum nigrum* associated with the fresh leaves of *E. abyssinica* Steud. Ex A. Rich., Fabaceae, exhibited significant ABTS and DPPH scavenging activities with an IC_50_ of 38.43 ± 4.85 and 49.45 ± 6.52 µg/mL, respectively [30].

Phomopsol B (**247**) and **248** were identified in *Phomopsis* sp. Xy21. Compound **248** was composed of a pair of epimers of 3-(2,6-dihydroxyphenyl)-4-hydroxy-6-methyl-isobenzofuran-1(3H)-one at C-9 and possessed neuroprotection, improving cell viability by 96% for corticosterone-mediated PC12 cell damage at 40.0 μM, whereas **247** did not display any such activity within the scope of 5.0−40.0 μM [38].

Three new p-terphenyls, 6′-*O*-Odesmethylterphenyllin (**249**), 3-hydroxy-6′-*O*-desmethylterphenyllin (**250**), and 3″-deoxy-6′-*O*-desmethylcandidusin B (**252**), along with two known *p*-terphenyls, 3,3″-dihydroxy-6′-*O*-desmethylterphenyllin (**251**) and 6′-*O*-desmethylcandidusin B (**253**), were collected from *Penicillium chermesinum* (ZH4-E2) associated with *Kandelia candel*. Compounds **252** and **253** inhibited AChE with IC_50_s of 7.8 and 5.2 μM, respectively. The other compounds did not exhibit AChE inhibition with an IC_50_ beyond 100 μM [57].

Chemical research on the endophytic *Chaetomium globosum* isolated from the seeds of *Panax notoginseng* resulted in the identification of flavipin (**254**), epicoccone (**255**), 3-methoxyepicoccone (**256**), and epicoccolides A (**257**) and B (**258**). Compound **256** possessed anti-AChE activity with an inhibition ratio of 72.6% at 50 μM. Compound **258** displayed obvious inhibitory activity against AChE with an IC_50_ of 5.55 μM. The AChE inhibition rates of **254**, **255**, and **257** were lower than 10% at 50 μM. The structure–activity relationship revealed that the key group for AChE inhibition was an oxygenic five-membered ring between **256** and **258 [17]**.

A new enalin analog, 7-hydroxy-2,4dimethyl-3(2H)-benzofuranone (**263**), together with five known compounds, including butyrolactone I (**259**), ulocladol diacetate (**260**), ulocladol triacetate (**261**), 2,5-diacetylphenol (**262**), and enalin [2,7-dihydroxy-2,4-dimethyl-3(2H)-benzofuranone] (**264**), were isolated from *Microsphaeropsis olivacea* related to *Pilgerodendron uviferum* (D. Don) Florin (“Cipres de las Guaitecas”). The IC_50_s of **260**–**262** for AChE inhibition were 83, 37, and 89 µg/mL, respectively [52].

An intensive chemical assay for *Corynespora cassiicola* L36 from *Lindenbergi philippensis* (Cham.) resulted in the observation of corynesidones A (**265**) and B (**266**), corynether A (**267**), and a diaryl ether (**268**). Corynesidone B (**266**) showed scavenging DPPH activity with IC_50_ = 22.4 µM [63].

An investigation of *Penicillium citrinum* from *Bruguiera gymnorrhiza* led to the identification of (Z)-7,40-dimethoxy-6-hydroxy-aurone-4-O-b-glucopyranoside (**269**) and (1S,3R,4S)-1-(40-hydroxyl-phenyl)-3,4-dihydro-3,4,5-trimethyl-1H-2-benzopyran-6,8-diol (**270**). Compound **269** showed stronger neuroprotection than did **270** with respect to MPP^+^-mediated PC12 cell damage. The mechanism of **269** involved improving cell viability and mitochondrial membrane potential, inhibiting caspase-3 and caspase-9 expression and reducing DNA fragment formation [64].

The isobenzofuranone isopestacin (**271**) was identified in the endophytic fungus *Pestalotiopsis microspora* isolated from *Terminalia morobensis*. Compound **271** exhibited potent scavenging OH activity at 0.22 mM [65].

Oosporein (**272**) identified in the endophyte *Cochliobolus kusanoi* from *Nerium oleander* L demonstrated a 50% scavenging DPPH capacity at 0.194 mM [66].

**Figure 11:**
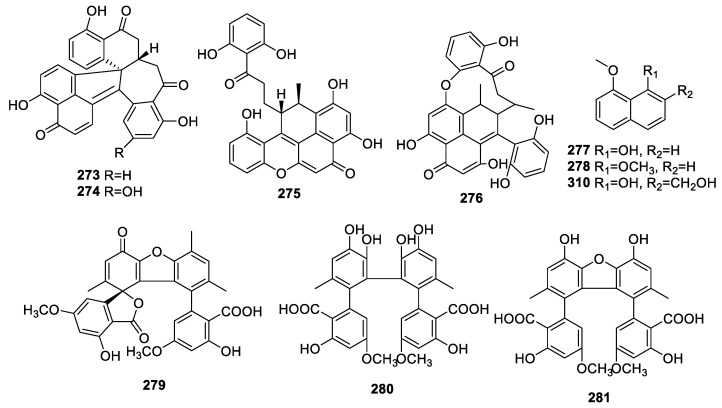
Chemical structures of other polyketides (**273**–**281**, **310**).

The careful chemical study of *Sporothrix* sp. (#4335) revealed the isolation of sporothrins A–C (**273**–**275**) and sporothrin C (**276**), 1-hydroxy 8-methoxy-naphthalene (**277**), and 1,8-dimethoxy-naphthalene (**278**) (Figure 11). Compound **253** showed potent AChE inhibition with IC_50_ at 1.05 μM [67,68].

Three novel aromatic polyketide dimers, bialternacins B–D (**279**–**281**), were collected from *Alternaria sp.* interrelated with the stem of *Maianthemum bifolium*. Compound **281** alone exhibited anti-AChE capacity with IC_50_ at 68.3 μM [56].

**Figure 12:**
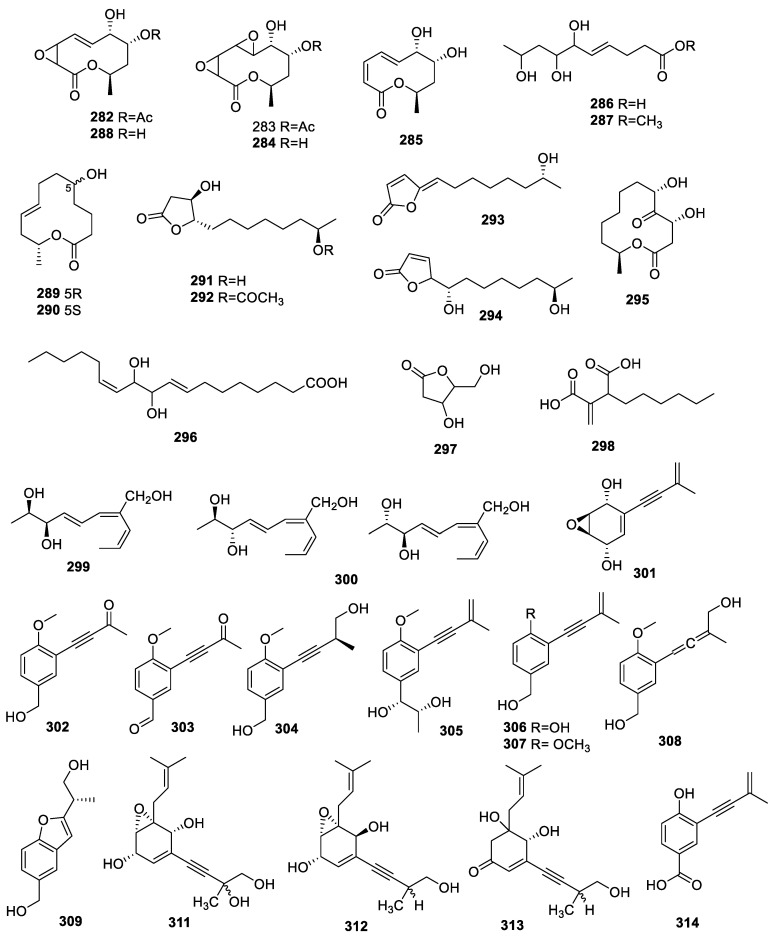
Chemical structures of other polyketides **282**–**309**, **311**–**314**.

The investigation of *Phomopsis* sp. NXZ-05 related to the twigs of *Camptotheca acuminata* DECNE. (Nyssaceae). revealed seven compounds: 8-O-acetylmultiplolide A (**282**), 8-O-acetyl-5,6-dihydro-5,6-epoxymultiplolide A (**283**), 5,6-dihydro-5,6-epoxymultiplolide A (**284**), 3,4-deoxy-3,4-didehydromul-tiplolide A (**285**), (4E)-6,7,9-trihydroxydec-4-enoic acid (**286**), methyl (4E)-6,7,9-trihydroxydec-4-enoate (**287**), and multiplolide A (**288**) (Figure 12). The evaluation of AChE inhibition for **282**–**284** and **288** indicated that **282** possessed obvious anti-AChE activity with an IC_50_ of 1.19 mg/mL, while the other compounds exhibited no apparent activity with an IC_50_ beyond 10 mg/mL [69].

Detailed chemical research on *Cladosporium cladosporioides* MA-299, an endophytic fungus from the mangrove plant leaves of *Bruguiera gymnorrhiza*, contributed to the isolation of new compounds 5*R*-hydroxyrecifeiolide (**289**), 5*S*-hydroxyrecifeiolide (**290**), ent-cladospolide F (**291**), cladospolide G (**292**), and cladospolide H (**293**) together with known compounds iso-cladospolide B (**294**) and pandangolide 1 (**295**). Among them, **291** alone exhibited strong AChE inhibition with an IC_50_ value of 40.26 µM [70].

A study on *Aspergillus flavus* cf-5 from the red alga *Corallina officinalis* revealed the isolation of the new compound (8*E*,12*Z*)-10,11-dihydroxyoctadeca-8,12-dienoic acid (**296**), which had a weak AChE inhibitory capacity with a rate of 10.3% at 100 µg/mL [71].

2′-Deoxyribolactone (**297**) and hexylitaconic acid (**298**) were identified from a new endophyte *Curvularia* sp., which was discovered on the stem bark of *Rauwolfia macrophylla*. The IC_50_s of **297** and **298** for inhibiting AChE were 1.93 and 1.54 μM, respectively [72].

A chemical assay for *Talaromyces aurantiacus* demonstrated the separation of two new compounds: talaromycins A (**299**) and B (**300**). The IC_50_ of **299** for AChE inhibition was 12.63 μM [73].

The compound E-G6-32 (**301**) was isolated from the endophyte *Curvularia* sp. G6-32 from the plant *Sapindus saponaria* L. It showed anti-DPPH and anti-ABTS activities with inhibitory rates of 22.5% and 62.7%, respectively [74].

The extensive investigation of *Daldinia* sp. TJ403-LS1 collected from *Anoectochilus roxburghii* led to the identification of five new acetylenic phenol derivatives, daldiniols A–E (**302**–**305**, **308**); one new benzofuran derivative, daldiniol F (**309**); one new naphthol derivative, daldiniol G (**310**); and two known analogs, 4-hydroxy-3-(3-methylbut-3-en-l-ynyl)benzyl alcohol (**306**) and methoxy-3-(3-methylbut-3-enl-ynyl)benzyl alcohol (**307**). The IC_50_s of **306**, **307**, **309**, and **310** for anti-BChE activities were 6.93 ± 0.71, 16.00 ± 0.30, 23.33 ± 0.55, and 15.53 ± 0.39 μM, respectively [75].

Three new oxygenated cyclohexanoids, speciosins U–W (**311**–**313**), along with 4-hydroxy-3-(3′-methylbut-3′-en-1′-ynyl)-benzoic acid (**314**) and 4-hydroxy-3-prenyl-benzoic acid (**315**), were reported in the *Saccharicola* sp. of *Eugenia jambolana*. Compound **311** alone exhibited inhibition toward huAChE-ICER and eeAChE-ICER with IC_50_s of 0.076 ± 0.01 and 0.0047 ± 0.0009 mg/mL, respectively [58].

**Figure 13:**
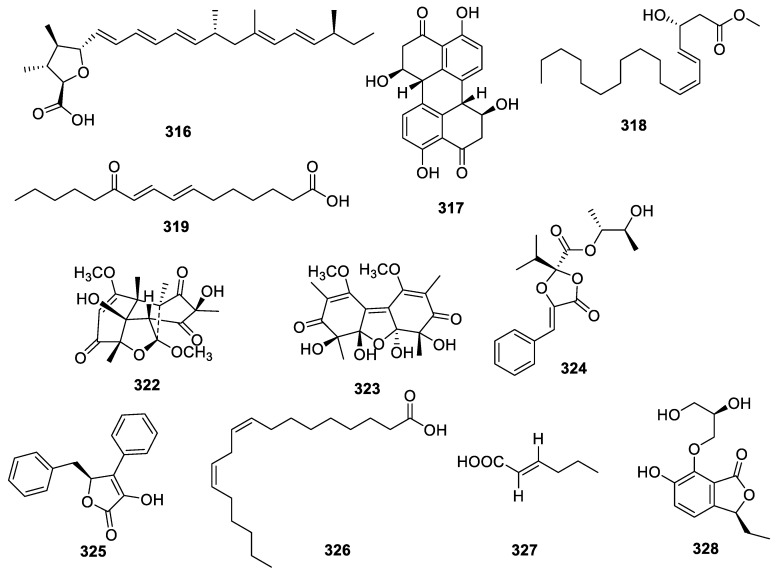
Chemical structures of other polyketides (**316**–**319**, and **322**–**328**).

A comprehensive assay for *Alternaria alternate* from the leaves of *Psidium littorale* Raddi resulted in the discovery of a new liphatic polyketone, alternin A (**316**), as well as the known compounds stemphyperylenol (**317**), 3(ζ)hydroxy-octadeca-4(*E*),6(*Z*)-dienoic acid (**318**), *E*-7,9-diene-11-methenyl palmitic acid (**319**), *p*-hydroxybenzonic acid (**320**), and benzoic acid (**321**) (Figure 13). Compound **316** exhibited a significant neuroprotective capacity against glutamate-induced PC12 cell death, with cell viabilities improving from 64.7 ± 4.9% to 72.3 ± 4.5% after treatment with 20, 40, and 80 μM [29].

Two unusual dimers, trematosphones A (**322**) and B (**323**), were separated from the endophyte *Trematosphaeria terricola* isolated from desert plant *Artemisia desertorum*. Compound **322** alone dispalyed neuroprotection for corticosterone-induced PC12 cell damage at 6.25 μM [76].

A study on *Phyllosticta capitalensis* from the leaves of *Loropetalum chinense* var. rubrum led to the isolation of the new compound guignardianone G (**324**), together with three known compounds: xenofuranone B (**325**), linoleic acid (**326**), and 2-hexenoic acid (**327**). Compound **326** showed potential neuroprotective activities toward glutamate-injured PC12 cells with an EC_50_ of 33.9 μM. Compound **324** showed no neuroprotective activity at 40 μM, and **325** and **327** even exhibited weak cytotoxicity at 40 μM [77].

A phthalide glycerol ether (**328**) was found in *Cochliobolus lunatus* SCSIO41401. This compound displayed mild AChE inhibition with IC_50_ at 2.5 ± 0.21 μM, while the IC_50_ for the active control of huperzine A was 0.30 ± 0.06 μM [40].

**Figure 14:**
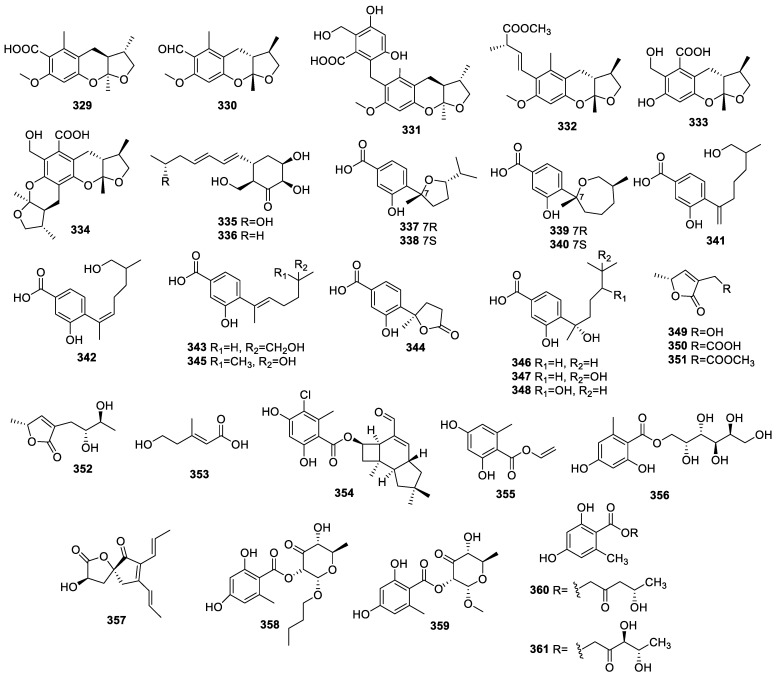
Chemical structures of other polyketides (**329**–**361**).

Phomeketales A–F (**329**–**334**) (Figure 14) were separated from *Phoma* sp. YN02-P-3. Compound **331** alone exhibited moderate AChE inhibition with IC_50_ at 40.0 μM [78].

Extensive research on *Penicillium* sp. sk14JW2P collected from the roots of *Kandelia candel* (L.) DRUCE revealed the existence of 13-hydroxypalitantin (**335**) and (+)-palitantin (**336**), which exhibited anti-AChE activities with IC_50_ values of 12 ± 0.3 and 79 ± 2 nM, respectively, while the IC_50_ for the positive control of huperzine A was 0.06 μM [79].

The intensive study of endophyte *Aspergillus* sp. xy02 from a Thai mangrove *Xylocarpus moluccensis* uncovered seven new compounds, including (7R,10S)-7,10-epoxysydonic acid (**337**), (7S,10S)-7,10-epoxysydonic acid (**338**), (7R,11S)-7,12-epoxysydonic acid (**339**), (7S,11S)-7,12-epoxysydonic acid (**340**), 7-deoxy-7,14-didehydro-12-hydroxysydonic acid (**341**), (Z)-7-deoxy-7,8-didehydro-12-hydro-xysydonic acid (**342**), and (E)-7-deoxy-7,8-didehydro-12-hydroxysydonic acid (**343**), as well as five known compounds: (+)-1-hydroxyboivinianic acid (**344**), engyodontiumone I (**345**), (+)-sydonic acid (**346**), (+)-hydroxysydonic acid (**347**), and (−)-(7S)-10-hy-droxysydonic acid (**348**). Compound **348** alone displayed a moderate scavenging DPPH capacity with an IC_50_ of 72.1 μM [80].

Intensive chemical research on *Phaeosphaeria* sp. LF5 from the leaves of *Huperzia serrata* generated the identification of 3-(hydroxymethyl)-5-methylfuran-2(5H)-one (**349**), aspilactonols G–I (**350**–**352**), and E-∆^2^-anhydromevalonic acid (**353**). Compound **352** alone exhibited anti-AChE activity with IC_50_ at 6.26 µM. The other compounds showed no activity at 100 µM [49].

Investigation into a co-culture of endophyte *Epicoccum sp.* YUD17002 and *Armillaria sp*. contributed to the discovery of armilliphatics A–C (**354**–**356**). The IC_50_ value of compound **354** for anti-AChE activity was 23.85 μM. The other compounds were inactive against AChE at 50 μM [81].

A rare 1-oxaspiro chaetospirolactone (**357**), orsellide F (**358**), orsellide A (**359**), globosumone B (**360**), and globosumone C (**361**) were obtained from *Chaetoium* sp. NF00754. The IC_50_ values for compounds **359** and **361** for anti-AChE activity were 7.34 and 7.67 µM, respectively [82].

### 2.4. Terpenoids

#### 2.4.1. Sesquiterpenoids

**Figure 15:**
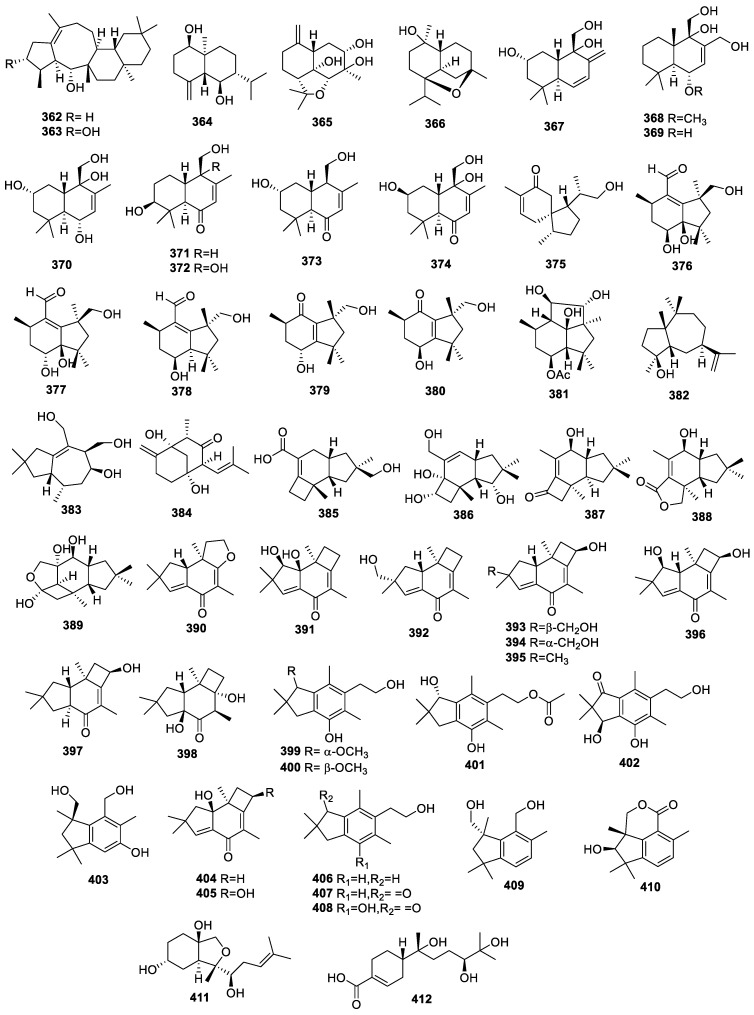
Chemical structures of sesquiterpenoids (**362**–**412**).

Two new compounds, asperterpenols A (**362**) and B (**363**) (Figure 15), with a rare 5/8/6/6 tetracyclic ring skeleton, were separated from *Aspergillus* sp. 085242. Compounds **362** and **363** powerfully inhibited AChE with IC_50_s of 2.3 and 3.0 μM, respectively. Neither compound inhibited BChE (IC_50_ >100 μM) [83].

The new compound (1*R*,5*R*,6*R*,7*R*,10*S*)-1,6-dihroxyeudesm-4(15)-ene (**364**) was identified from *Alternaria alternate* interrelated with the leaves of *Psidium littorale* Raddi. This compound was inactive for neuroprotective activity toward glutamate-injured PC12 cells at 40 and 80 μM [29].

The extensive chemical investigation of endophyte *Paecilomyces* sp. TE-540 associated with the fresh leaves of *Nicotiana tabacum* L. led to the identification of two new cadinane-type sesquiterpenes, paecilacadinols A (**365**) and B (**366**), and two new drimane-type sesquiterpenes, ustusol D (**367**) and ustusol E (**368**), along with known compounds 12-hydroxyalbrassitriol **(369**), 2-hydroxyalbrassitriol (**370**), deoxyuvidin B (**371**), 3*β*,9*α*,11-trihydroxy-6-oxodrim-7-ene (**372**), 2*α*,11-dihydroxy-6-oxodrim-7-ene (**373**), and ustusol B (**374**). The AChE inhibition ratios of **365**–**374** were in the range of 17.56 ± 3.33 to 57.38 ± 4.51%. The IC_50_s of **369** and **370** for anti-AChE activities were 43.02 ± 6.01 and 35.97 ± 2.12 μM, respectively. The binding sites of **369** to the AChE catalytic pocket were Trp84, Gly117, Ser122, and Tyr121 residues, while **370** lay on Asp72 and Ser122 residues [84].

A study on *Pseudofusicoccum* sp. J003 from the mangrove species *Sonneratia apetala* Buch.-Ham led to the separation of the new sesquiterpene, acorenone C (**375**), which exhibited moderate activity against AChE with a 23.34% inhibition ratio at 50 μM [85].

Comprehensive research on *Nemania bipapillata* (AT-05) from the marine red alga *Asparagopsis taxiformis-Falkenbergia* stage led to the discovery of (+)-(2R,4S,5R,8S) (**376**), (+)-(2R,4R,5R,8S)-4-deacetyl-5-hydroxy-botryenalol (**377**), (+)-(2R,4S,5R,8R)-4-deacetyl-botryenalol (**378**), (+)-(2R,4R,8R) (**379**), (+)-(2R,4S,8S)-(**380**), and 4β-acetoxy-9β,10β,15α-trihydroxyprobotrydial (**381**). Compounds **376**–**381** showed AChE and BChE inhibition with inhibitory ratios of 18.3% and 27.7%, and 3.2% and 7.3% at 100 μM, respectively [86].

Guaidiol (**382**) was identified in *Xylaria* sp. HNWSW-2. The inhibition rate of **382** against AChE was 12.9% at 50 µg/mL [46].

Nigrosirpexin A (**383**) was collected from a co-culture of *Nigrospora oryzae* and *Irpex lacteus.* This compound showed an AChE inhibitory capacity with a ratio of 35% at 50 µM [87].

A chemical assay for *Colletotrichum gloeosporioides* GT-7 from the healthy tissue of *Uncaria rhynchophylla* produced colletotrichine A (**384**), which inhibited AChE with IC_50_ at 28 μg/mL [88].

A co-culture of *Armillaria sp.* and endophyte *Epicoccum sp.* generated five protoilludane-type sesquiterpenoids, epicoterpenes A−E (**385**–**389**), which were inactive for AChE inhibition at 50 μM [47].

The comprehensive chemical investigation of *Phomopsis* sp. TJ507A from *Phyllanthus glaucus* led to the identification of a 2,3-*seco*-protoilludane-type sesquiterpene, phomophyllin A (**390**); eight protoilludane-type sesquiterpenes, phomophyllins B−I (**391**–**398**); four illudalane-type sesquiterpenes, phomophyllins J−M (**399**/**400**, **401**, and **402**); and a botryane-type sesquiterpene, phomophyllin N (**403**). In addition, seven known sesquiterpenoids, granulone B (**404**), radulone B (**405**), 2-(2,2,4,6-tetramethylindan-5-yl)ethanol (**406**), pterosin Z (**407**), onitin (**408**), dehydrobotrydienol (**409**), and 7-hydroxy-10-oxodehydrodihydrobotrydial (**410**), were also isolated from this fungus. This represents the first natural product of **390** with an irregular 2,3-*seco*-protoilludane skeleton. Compounds **390**–**396**, **398**, **405**, **408**, and **410** inhibited BACE1 within the range of 19.4% to 43.8% at 40 μM [89].

The fungus *Colletotrichum gloeosporioides* GT-7 generated the compound colletotrichine B (**411**), which inhibited AChE with IC_50_ at 38.0 ± 2.67 μg/mL [90].

A chemical assay for *Colletotrichum* sp. SCSIO KcB3-2 from *Kandelia candel* produced a new polychiral bisabolane sesquiterpene of bisabolanoic acid A (**412**), which exhibited mild AChE inhibition with an IC_50_ of 2.2 μM, whereas the IC_50_ for the positive control of huperzine A was 0.30 ± 0.06 μM [91].

#### 2.4.2. Meroterpenoids

**Figure 16:**
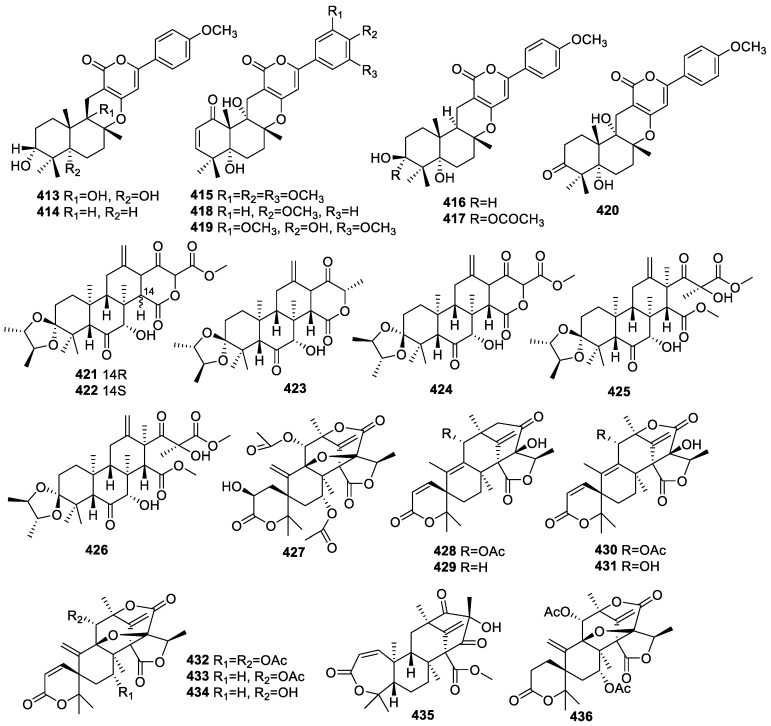
Chemical structures of meroterpenoids (**413**–**436**).

Extensive research on *Penicillium* sp. SK5GW1L, a mangrove endophytic fungus from the leaves of *Kandelia candel*, resulted in the separation of two new *α*-pyrone meroterpenoids, arigsugacin I (**413**) and 3-epiarigsugacin E (**416**), together with seven known analogs: arigsugacin F (**414**), territrem B (**415**), arisugacin D (**417**), arisugacin B (**418**), territrem C (**419**), and terreulactone C (**420**) (Figure 16). The IC_50_ values for all the isolates against AChE were 0.64, 0.37, 7.03, 38.23, 53.39, 3.03, 0.23, and 0.028 μM, respectively [92,93].

The investigation of *Aspergillus terreus* Thom, an endophytic fungus from *Tripterygium wilfordii* Hook. f. (Celastraceae), revealed six undescribed meroterpenoids, spiroterreusnoids A–F (**421**–**426**). The IC_50_s of **421**–**426** for BACE1 and AChE inhibition ranged from 5.86 to 27.16 μM and from 22.18 to 32.51 μM, respectively [94].

A detailed study on *Aspergillus* 16-5c, a mangrove endophytic fungus identified from *Sonneratia apetala*, found one new meroterpenoid, 2-hydro-acetoxydehydroaustin (**427**), along with known analogs 11-acetoxyisoaustinone (**428**), isoaustinol (**429**), austin (**430**), austinol (**431**), acetoxydehydroaustin (**432**), dehydroaustin (**433**), dehydroaustinol (**434**), preaustinoid A2 (**435**), and 1,2-dihydro-acetoxydehydroaustin B (**436**). The IC_50_s for AChE inhibition by compounds **429**, **433**, and **434** were 2.50, 0.40, and 3.00 µM, respectively [95].

#### 2.4.3. Diterpenoids

**Figure 17:**
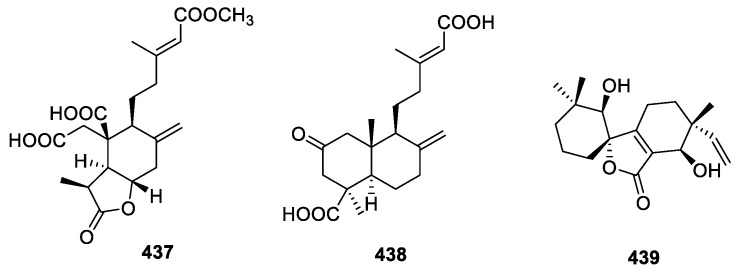
Chemical structures of diterpenoids (**437**–**439**).

Chemical research on *Penicillium chrysogenum* MT-12 collected from *Huperzia serrata* revealed the new compounds penicichrysogene A (**437**) and penicichryso-gene B (**438**) (Figure 17). Unfortunately, neither compound showed obvious AChE and BChE inhibition at 100 µM [96].

A study on the *Aspergillus* sp. YXf3 of *Ginkgo biloba* found an irregular C18 norditerpenoid, aspergiloid I (**439**), which did not exhibit antioxidant properties or AChE inhibition at 50 μg/mL [97].

### 2.5. Steroids

**Figure 18:**
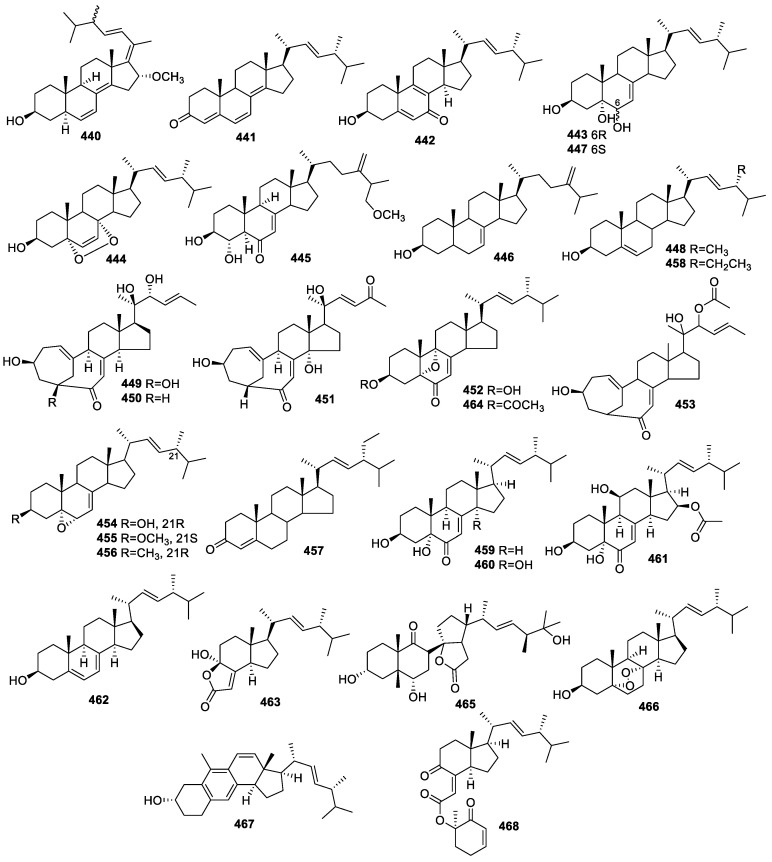
Chemical structures of steroids (**440**–**468**).

A new steroid, asporyergosterol (**440**), along with four known steroids, containing (22*E*,24*R*)-ergosta-4,6,8(14),22-tetraen-3-one (**441**), (22*E*,24*R*)-3β-hydroxyergosta-5,8,22-trien-7-one (**442**), (22*E*,24*R*)-ergosta-7,22-dien-3β,5α,6β-triol (**443**), and (22*E*,24*R*)-5α,8α-epidioxyergosta-6,22-dien-3β-ol (**444**) (Figure 18), were identified from culture extracts of *Aspergillus oryzae* associated with the marine red alga *Heterosiphonia japonica*. All the compounds exhibited a low capacity to modulate AChE with inhibitory rates from 0.4%–19.8% at 100 µg/mL [98].

The instentive investigation of *Aspergillus flavus* cf-5 from the marine red alga *Corallina officinalis* led to the separation of a new compound, 3*β*,4*α*-dihydroxy26-methoxyergosta-7,24(28)-dien-6-one (**445**), as well as four known isolates: episterol (**446**), (22*E*,24*R*)-ergosta7,22-dien-3*β*,5*α*,6*α*-triol (**447**), (22*E*,24*R*)-ergosta-5,22-dien-3*β*-ol (**448**), and (22*E*,24*R*)-ergosta-4,6,8(14),22-tetraen-3-one (**441**). Compound **445** displayed weak activity against AChE with an inhibition ratio of 5.5% at 100 µg/mL [71].

A study on *Chaetomium* sp. M453 associated with *Huperzia serrata* (Thunb. ex Murry) Trev produced the isolation of neocyclocitrinols E–G (**449**–**451**) and 3β-hydroxy-5,9-epoxy-(22*E*,24*R*)-ergosta-7,22-dien-6-one (**452**) as well as three known steroids (**453**–**455**) separated from the endophytic fungus *Chaetomium* sp. M453 associated with *Huperzia serrata* (Thunb. ex Murry) Trev. Compounds **451**–**452** were assayed for AChE inhibitory activities. Compound **452** alone showed weak AChE inhibitory activity at 50 μM [99].

Four known steroids, (3β,5α,6α, 22*E*)-3-hydroxy-5,6-epoxy7-one-8(14),22-dien-ergosta (**456**), **443**, β-sitostenone (**457**), and β-sitosterol (**458**), and **448** were obtained from *Chaetomium* sp. YMF432 related to *Huperzia serrata* (Thunb. ex Murray) Trev. Compound **456** alone showed moderate AChE inhibition with an IC_50_ of 67.8 ± 1.7 μM and an inhibitory rate of 58.8 % at 100 μg/mL [60].

An extensive study on *Aspergillus terreus* (No. GX7-3B) from a branch of *Bruguiera gymnoihiza* (Linn.) Savigny resulted in the separation of 3β,5α-dihydroxy-(22*E*,24*R*)-ergosta-7,22-dien-6-one (**459**), 3β,5α,14α-trihydroxy-(22*E*,24*R*)-ergosta-7, 22-dien-6-one (**460**), and NGA0187 (**461**). Compound **461** displayed remarkable anti-AChE activity with an IC_50_ value of 1.89 μM [36].

Ergosterol (**462**) was identified from *Curvularia* sp. associated with *Rauwolfia macsrophylla*. The IC_50_ of **462** for AChE inhibitory activity was 1.52 μM [72].

Two known steroids, **441** and (17*R*)-4-hydroxy-17-methylincisterol (**463**), were identified from *Alternaria alternate* related to the leaves of *Psidium littorale* Raddi. Compounds **441** and **463** were inactive for neuroprotective activity toward glutamate-injured PC12 cells at 40 and 80 μM, respectively [29].

Research on *Colletotrichum* sp. F168 from the plant *Huperzia serrata* Trev produced the compound ergosta-7,22-dien-5,9-epoxy-(22E,24R)-6-one-3-yl acetate (**464**), which showed a negligible AChE inhibitory activity of 18.2% at 100 µg/mL [62].

The investigation of *Talaromyces* sp. SCNU-F0041 from the fresh leaves of *Kandelia* produced cyclosecosteroid A (**465**), ergosterol (**462**), (22E,24R)-5α,8α-epidioxyergosta-6,22-dien-3β-ol (**466**), and cerevisterol (**443**). The IC_50_ of compound **465** for inhibiting AChE was 46 μM [100].

Brassicasterol (**448**), 5,6-epoxyergosterol (**454**), citreoanthrasteroid A (**467**), demethylincisterol A (**463**), and chaxine C (**468**) were identified in *Phyllosticta capitalensis* derived from the leaves of *Loropetalum chinense* var. rubrum. Compound **467** alone exhibited neuroprotection with an EC_50_ of 24.2 μM for glutamate-mediated PC12 cell injury [77].

## 3. Conclusions

**Figure 19:**
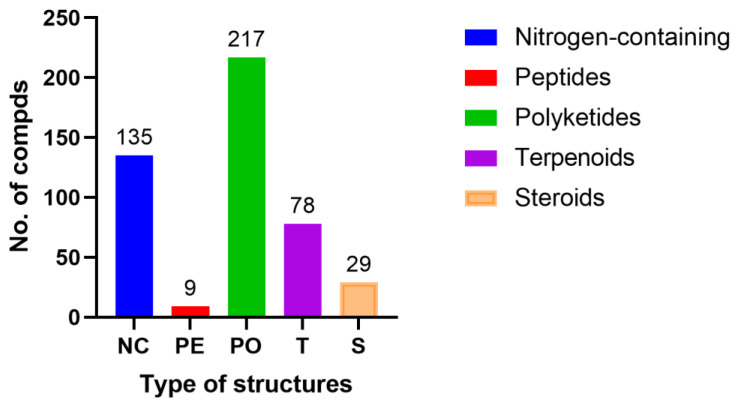
Different classes of metabolites reported in this review.

**Figure 20:**
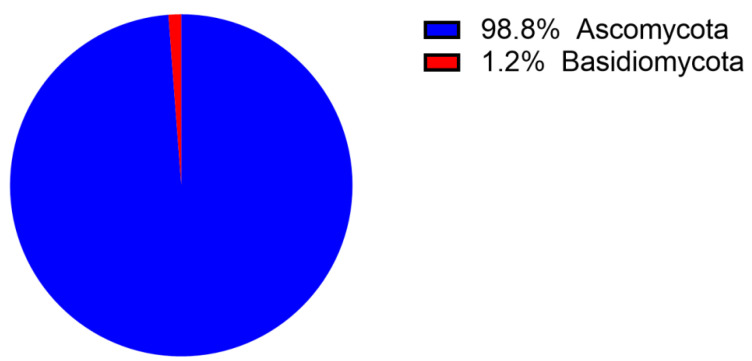
Taxonomy of endophytic fungi isolated from 2002–2022.

**Figure 21:**
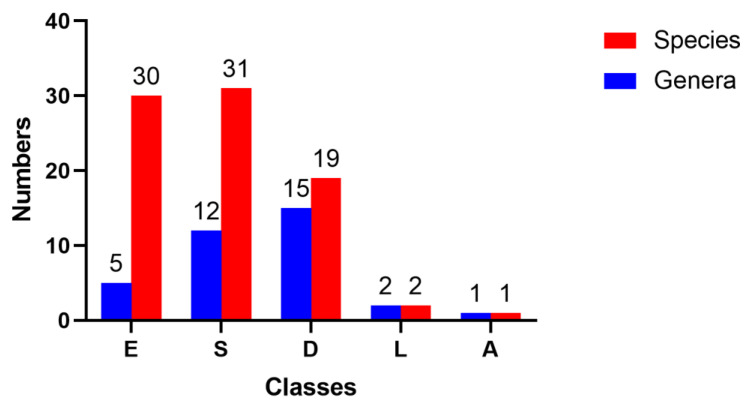
Taxonomy of endophytic fungi isolated from 2002–2022.

Endophytic fungi are significant treasured natural products that provide numerous bioactive compounds for the research of new drugs. According to the statistical results (Tables S1–S5, Figure 1, Figure 2, Figure 3, Figure 4, Figure 5, Figure 6, Figure 7, Figure 8, Figure 9, Figure 10, Figure 11, Figure 12, Figure 13, Figure 14, Figure 15, Figure 16, Figure 17 and Figure 18), 468 metabolites with anti-AD-related activities and diverse structural features were identified in this study. These isolated natural products from endophytes possessed diverse structural features and included alkaloids (135, 28.8%), peptides (9, 1.9%), polyketides (217, 46.4%), terpenoids (78, 16.7%), and steroids (29, 6.2%) (Figure 19). Among these compounds, polyketides were the most common, followed by alkaloids, terpenoids, and steroids. A total of 468 compounds were isolated from 83 endophytes, which were assigned to 2 phyla, 5 classes, and 35 genera. Taxonomically, nearly all the strains belonged to the phyla Ascomycotina (98.8%), including the classes Eurotinomycetes (36.1%), Sordariomycetes (37.3%), Dothideomycetes (22.9%), and Leotiomycetes (2.4%), while only Agaricomycetes belonged to the phylum Dasidiomycota (1.2%) (Figure 20). Some genera contained two or more species of endophytes that possess promising bioactive anti-Alzheimer’s components, including *Aspergillus* (13), *Penicillium* (11), *Colletotrichum* (9), *Phomopsis* (5), *Talaromyces* (4), *Chaetomium* (4), *Alternaria* (2), *Epicoccum* (2), *Cochliobolus* (2), and *Curvularia* (2) (Figure 21). Around 27.5% of the compounds were separated from the genera *Aspergillus* and *Penicillium*, accounting for 72 and 58 compounds, respectively.

Based on the analyzed data, the biological activity of these compounds was determined, mainly focusing on their anti-AChE, anti-BChE, antioxidant, and neurotrophic activities. Some of the compounds exhibited micromolar to nanomolar biological activities, such as chaetoglobosin F (**17**) and isochaetoglobosin D (**23**), which showed strong H_2_O_2_-induced PC12 cell damage-inhibiting activities with EC_50_s of 0.003 ± 0.0003 and 0.009 ± 0.001 μM, respectively. Huptremules C and D (**118**, **119**) showed stronger AChE-inhibiting activities, with IC_50_s of 0.11 ± 0.01 and 0.06 ± 0.00 μM, respectively, than hupA (IC_50_ = 0.54 μM). Hence, they represent valuable compounds for developing anti-AD agents. Notably, structural changes to these compounds directly affect their bioactivities. Synthesis and structural modifications for bioactive metabolites are necessary to prepare more effective analogs. This review confirmed the significance of endophytes in the generation of abundant metabolic products with anti-AD activities. In the future, with the addition of further in-depth research on endophytic fungal metabolites, more biologically active chemical resources will become available to medicinal chemists and biologists for anti-AD drug research.

## Data Availability

Not applicable.

## Supplementary Materials

The following supporting information can be downloaded at: https://www.mdpi.com/article/10.3390/molecules28052259/s1, Table S1: Alkaloids from endophytic fungi and their biological activities, metabolite class, fungus, host plant(s), reference; Table S2: Peptides from endophytic fungi and their biological activities, metabolite class, fungus, host plant(s), reference; Table S3: Polyketides from endophytic fungi and their biological activities, metabolite class, fungus, host plant(s), reference; Table S4: Terpenes from endophytic fungi and their biological activities, metabolite class, fungus, host plant(s), reference; Table S5: Steroids from endophytic fungi and their biological activities, metabolite class, fungus, host plant(s), reference.

## Abbreviations

AD	Alzheimer’s disease
AChE	Acetylcholinesterase
BChE	Butyrylcholinesterase
BACE1	β-site amyloid precursor protein-cleaving enzyme 1
PC12	Rat pheochromocytoma cells
DPPH	2,2-Diphenyl-1-picrylhydrazyl
ABTS	2,2-Azino-bis-(3-ethylbenzthiazoline-6-sulfonic acid)
EC_50_	Half effective concentration
IC_50_	Half maximal inhibitory concentration
H_2_O_2_	Hydrogen peroxide
HT22	Mouse hippocampal cells
JNK	c-Jun N-terminal kinase
ERK	Extracellular signal-regulated kinase
huAChE-ICER	Immobilized capillary enzyme reactors
ROS	Reactive oxygen species
MAPK	Mitogen-activated protein kinases
ORAC	Oxygen radial absorbance capacity against ROO∙
MPP^+^	1-Methy-4-phenylpyridinium
