# Supplementary material for: Anti-Alzheimer’s Natural Products Derived from Plant Endophytic Fungi
Source: Molecules. 2023 Feb 28;28(5):2259. doi: 10.3390/molecules28052259 (PMC10005758; doi:10.3390/molecules28052259)
Supplement: Supplementary file 1 [file molecules-28-02259-s001.zip › Table S1. Alkaloids from endophytic fungi and their biological activities, metabolite class, fungus, host plant(s), reference.pdf]

Table S1. Alkaloids from endophytic fungi and their biological activities, metabolite class, fungus, host plant(s), reference.

Metabolite Class	Fungus	Host Plant(s)	Compounds Isolated	Biological Target	Biological Activity	Reference
Cytochalasans	*Xylaria* sp.	*Casearia sylvestris*	Cytochalasins B (**1**)	AChE inhibitory activity	Potent AChE inhibition at 60 μg	13
			Cytochalasin C (**2**)			
			Cytochalasin D (**3**)		Inactive	
	Aspergillus terreus	*Artemisia annua*	Cytochalasin E (**4**)	AChE inhibitory activity	IC_{50}, 146.1±6.5, 176.0±11.5, and 110.9±13.7 μM, respectively	14
			5,6-Dehydro-7-hydroxy cytochalasin E (**5**)			
			Δ6,12-Isomer of 5 (**6**)			
			Rosellichalasin (**7**)		IC_{50} ＞ 200 μM	
	Phomopsis sp.	*Senna spectabilis* (Fabaceae)	Cytochalasins J (**8**)	AChE inhibitory activity	Inactive	15
	Phomopsis sp., *Phomopsis* sp. IFB-E060	*Senna spectabilis* (Fabaceae), *Vatica mangachapoi*	Cytochalasins H (**9**)	AChE inhibitory activity	Minimum quantity of 25.0 μg	15, 18
	Aspergillus flavipes	*Hevea brasiliensis*	Bisaspochalasin D (**10**)	Neurite growth activity of PC12 cells	Inducing a differentiation rate of 12.52% at 10 μM	16
			Bisaspochalasin E (**11**)		Inactive	
			Aspochalasin D (**12**)			
			Aspochalasin B (**13**)			
	Chaetomium globosum	*Panax notoginseng*	Chaetoglobosin A (**14**)	AChE inhibitory activity	Inhibition rate < 10% at 50 μM	17
			Chaetoglobosin B (**15**)		Inactive	
			Chaetoglobosin E (**16**)			
			Chaetoglobosin F (**17**)			

			Chaetoglobosin Fex (**18**)			
			Penochalasin F (**19**)			
			Penochalasin G (**20**)			
	Chaetomiun globosum WQ	*Imperata cylindrical*	Cytoglobosin A (**21**)	Scavenging DPPH and ABTS activity, H_2O_2-induced PC12 cell damage inhibitory activity	EC_{50}, 0.879 mM, 472.87 mM, and 0.023 mM, respectively	18
			Penocha-lasin C (**22**)		E_{max}, 7.1%, 5.9% and 40.3%, respectively	
			Isochaetoglobosin D (**23**)		EC_{50}, 0.002 mM, 0.002 mM, and 0.009 mM, respectively	
	Phomopsis sp. IFB-E060	*Vatica mangachapoi*	18-Methoxycytochalasin J (**24**)		EC_{50}: 481.121 mM, 1.052 mM, and 0.004 mM, respectively	
	Westerdykella nigra	*Avicennia marina* (Forssk.) Vierh	Westalsan (**25**)	AChE inhibitory activity	IC_{50}, 0.056±0.003, 0.088±0.005 and 0.140±0.007 μM, respectively	19
			Phomacin B (**26**)			
			19-Hydroxy-19,20-dihydro phomacin C (**27**)			
Diketopiperazine Derivatives	*Acrostalagmus luteoalbus* TK-43	*Codium fragile*	Acrozine A (**28**, **29**)	AChE inhibitory activity	IC_{50}, 9.5, 60.7 and 130.5 μM, respectively	20
			Acrozine B (**30**, **31**)			
			Acrozine C (**32**, **33**)			
			Acrozine D (**34**)		Weak activity at 32 μM	21
			Acrozine E (**35**)			
			Acrozine F (**36**)		IC_{50}, 8.4 μM	
			Acrozine G (**37**)		Weak activity at 32 μM	
			Pseudellone D (**38**)	–	–	
			Lasiodipine E (**39**)			
			Chetoseminudin C (**40**)			

		Chetoseminudin B (**41**)			
		T988 C (**42**)			
		T988 B (**43**)			
Aspergillus tetteus	*Artemisia annua*	Acetylapoaranotin (**44**)	AChE inhibitory activity	IC_{50}, 127.4±17.3μM	14
Neosartorya fischeri JS0553	*Glehnia littoralis*	Cyclotryprostatin B (**45**)	Neuroprotective activity against glutamate-mediated HT22 cell death.	Obvious neuroprotection at 20 μM	22
		Fumitremorgin B (**46**)			
		Fumitremorgin A (**47**)			
Talaromyces sp. LGT-2	*Tripterygium wilfordi*	Fumitremorgin C (**48**)	AChE inhibitory activity	Inactive	23
		Brevianamide F (**49**)			
		Spirotryprostatin A (**50**)			
		6-Methoxyspirotryprostatin B (**51**)			
		3-Dehydroxymethylbisdethio-3,10a-bis(methylthio)gliotoxin (**52**)			
		Bisdethiobis(methylthio)gliotoxin (**53**)		Weak activy	
		Didehydrobisdethiobis(methylthio)gliotoxin (**54**)	–	–	
Nigrospora camelliae-sinensis S30	*Lumnitzera littorea*	Nigrosporaamide A (**55**)	H_2O_2-mediated cytotoxicity for HT22 cell	Inactive at 100 μM	24
		Nigrosporaamide B (**56**)			
		Cyclo-(L-Pro-L-Phe) (**57**)			
		Cyclo[L-(4-hydroxyproliny			

			l)-L-Leu] (**58**)			
	Nigrospora camelliae-sinensis S30, *Penicillium* sp.1	*Lumnitzera littorea*, *Alibertia macrophylla* (Rubiaceae)	Cyclo-(L-Val-L-Pro) (**59**)	AChE inhibitory activity	A detection limit of 10.0 μg	24, 25
	Nigrospora camelliae-sinensis S30	*Alibertia macrophylla* (Rubiaceae)	Cyclo-(L-Leu–L-Pro) (**60**)	H_2O_2-mediated cytotoxicity for HT22 cell	Inactive at 100 μM	24
			Cyclo-(R-Leu-R-Pro) (**61**)			
			Cyclo-(L-Ile-L-Pro) (**62**)			
			Cyclo-(4-methyl-R-Pro-S-Nva) (**63**)			
	Colletotrichum gloeosporioides	*Michelia champaca*	Cyclo-(S-Pro-S-Tyr) (**64**)	AChE inhibitory activity	Moderate activity at 200 μg	26
			Cyclo-(S-Pro-S-Val) (**65**)			
	Colletotrichum crassipes	*Casearia sylvestris*	Cyclo(D)-Pro-(L)-Val (**66**)	AChE inhibitory activity	Inactive at 100 μg	13
			Cyclo(D)-Pro-(D)-Tyr (**67**)			
			Cyclo(D)-Val-(D)-Tyr (**68**)			
			Cyclo(D)-Hyp-(L)-Ile (**69**)			
			Cyclo(D)-Pro-(D)-Leu (**70**)			
			Cyclo(D)-Pro-(L)-Ile (**71**)			
			Cyclo(D)-Pro-(L)-Phe (**72**)			
			Cyclo(D)-Pro-(D)-Phe (**73**)		Potent activity at 60 μg	
Indole Alkaloids	*Aspergillus terreus*	*Artemisia annua*	16α-Hydroxy-5N-acetylardeemin (**74**)	AChE inhibitory activity	IC_{50}, 58.3±3.3, 149.4±11.3, and 116.9±3.7 μM, respectively	14
			5N-Acetylardeemin (**75**)			
			15b-β-Hydroxy-5N-acetylardeemin (**76**)			
	Colletotrichum	*Michelia champaca*	2-Phenylethyl	AChE inhibitory activity	Moderate activity at 200 μg	26

	gloeosporioides		1H-indol-3-yl-acetate (**77**)			
	Penicillium chrysogenum No. 005	*Cistanche deserticola*	Chrysogenamide A (**78**)	Neuroprorection for H_2O_2-mediated SH-SY5Y cell death	Enhancing cell viability of 59.6% at 1×10^{-4} μM	27
	Aspergillus fumigatus	*Cynodon dactylon*	9-Deacetylfumigaclavine C (**79**)	Neurite outgrowth of PC12	Inactive	28
			9-Deacetoxyfumigaclavine C (**80**)			
			Fumigaclavine C (**81**)			
	Neosartorya fischeri JS0553	*Glehnia littoralis*	Aszonalenin (**82**)	Neuroprotection against glutamate-mediated HT22 cell death	Inactive	22
			Acetylaszonalenin (**83**)			
	Alternaria alternate	*Psidium littorale* Raddi	Alternatine A (**84**)	PC12 cell injury induced by glutamate	Inactive	29
	Alternaria. Alternate/Epicoccum nigrum	*Psidium littorale* Raddi/ *Houttuynia cordata*	1*H*-indole-3-carboxylic acid (**85**)	Scavenging DPPH and ABTS activity	IC_{50}, 88.97 μg/mL for DPPH, EC_{50}, 21.48 ± 0.88 μM for ABTS	29,30,31
	Alternaria alternate	*Psidium littorale* Raddi	Indole-3-methylethanoate (**86**)	Neuroprotection on glutamate-mediated PC12 cell death	Cell viabilities improved with 75.6 ± 4.2% and 84.8 ± 6.5 at 40 and 80 μM	29
	Colletotrichum sp. SC1355	*Cleistocalyx operculatus*	Colletotryptin A (**87**)	AChE inhibitory activity	Inactive at 50 μM	32
			Colletotryptin B (**88**)			
			Colletotryptin C (**89**)			
			Colletotryptin D (**90**)			
			Colletotryptin E (**91**/**92**)			
			Colletotryptin F (**93**)			

Other Types of Alkaloids	*Colletotrichum gloeosporioides*	*Michelia champaca*	Uracil (**94**)	AChE inhibitory activity	Moderate activity at 200 μg	26
			4-Hydroxy-benzamide (**95**)			
	Botryosphaeria dothidea KJ-1	*Melia azedarach* L	3-Hydroxy-2-methoxy-5-methylpyridin-2(1H)-one (**96**)	Scavenging DPPH activity	Scavenging rate of 22.5% at 50 μM	33
	Nigrospora camelliae-sinensis S30	Lumnitzera littorea	5-(40-Hydroxybenzyl) hydantoin (**97**)	H_2O_2-mediated cytotoxicity for HT22 cell	Inactive	24
	Penicillium steckii SCSIO 41025	*Avicennia. Marina* (Forsk.) Vierh (Trichomaceae)	(±)-Oxypenicinoline A (**98/99**)	AChE inhibitory activity	Inactive	34
			(±)-Oxypenicinoline B (**100/101**)			
			(±)-Oxypenicinoline C (**102/103**)			
			(±)-Oxypenicinoline D (**104/105**)			
			Penicinoline F (**106**)			
			Penicinoline G (**107**)			
			1,2,3,11b-Tetrahydroquinolactacide (**108/109**)			
			Quinolactacide (**110**)			
			Penicinoline (**111**)		IC_{50}, 87.3 μM	
			Methyl-penicinoline (**112**)		Inactive	
			Penicinoline E (113)		IC_{50}, 68.5 μM	
			Quinolonimide (**114**)		Inactive	
			4-Oxo-1,4-dihydroquinolin			

		e-3-carboxamide (**115**)			
Ceriporia lacerate HS-ZJUT-C13A	*Huperzia Serrata*	Huptremule A (**116**)	AChE inhibitory activity	IC_{50}, 0.99±0.03, 2.17±0.05, 0.11±0.01, 0.06±0.00, and 12.11±0.34 μM, respectively	35
		Huptremule B (**117**)			
		Huptremule C (**118**)			
		Huptremule D (**119**)			
		8α,15α-Epoxyhuperzine A (**120**)			
Aspergillus terreus (No. GX7-3B)	*Bruguiera gymnoihiza*	8-O-methylbostrycoidin (**121**)	AChE inhibitory activity	IC_{50}, 6.71 μM	36
Fusarium sp. HP-2	Chinese agarwood "Qi-Nan"	Lumichrome (**122**)	AChE inhibitory activity	Inactive at 50 μM	37
Phomopsis sp. xy21	Thai *Xylocarpus granatum*	Phomopsol A (**123**)	Neuroprotection corticosterone-mediated PC12 cell injury	Cell viabilities of 76% at 40.0 μM	38
Aspergillus fumigatus	*Cynodon dactylon*	14-Norpseurotin (**124**)	Inducing neurite outgrowth	Promoting neurite outgrowth of PC12 at 10.0 μM	28
		Pseurotin A (**125**)			
Neosartorya fischeri JS0553	*Glehnia littoralis*	Fischerin (**126**)	Neuroprotection against glutamate-mediated HT22 cell death	100% cell viability at 5 μM	22
		Pyripyropene A (**127**)		Inactive	
Penicillium sp. JVF17	*Vitex rotundifolia*	Penazaphilone E (**128**)	Neuroprotection against glutamate-mediated HT22 cell death	Neuroprotective activities with almost 100% at 25 μM	39
		Isochromophilone VI (**129**)			
		Peniazaphilone D (**130**)			
Cochliobolus lunatus		Sinulariapeptide A (**131**)	AChE inhibitory activity	IC_{50}, 1.8 ± 0.12 and 1.3 ± 0.11	40

	SCSIO41401		Sinulariapeptide B (**132**)		μM, respectively	
	Rhizopycnis vagum Nitaf22	*Nicotiana tabacum*	Rhizovagine A (**133**)	AChE inhibitory activity	IC_{50}, 43.1 μM	41
	Talaromyces sp. LGT-2	*Tripterygium wilfordi*	Pseurotin A1 (**134**)	AChE inhibitory activity	Weaker	23
			Pseurotin A2 (**135**)			

"_" not test. IC_{50}, half maximal effective concentration; EC_{50}, half effective concentration; E_{max}, maximum effect; AChE, acethlcholinesterase; DPPH, 2,2-dipheny-1-picrylhydrazyl; ABTS, 2,2-azino-bis(3-ethylbenzothiazoline-6-sulfonic acid) diammonium salt.
