# Supplementary material for: Anti-Alzheimer’s Natural Products Derived from Plant Endophytic Fungi
Source: Molecules. 2023 Feb 28;28(5):2259. doi: 10.3390/molecules28052259 (PMC10005758; doi:10.3390/molecules28052259)
Supplement: Supplementary file 1 [file molecules-28-02259-s001.zip › Table S2. Peptides from endophytic fungi and their biological activities, metabolite class, fungus, host plant(s), reference.pdf]

Table S2. Peptides from endophytic fungi and their biological activities, metabolite class, fungus, host plant(s), reference.

Metabolite Class	Fungus	Host Plant(s)	Compounds Isolated	Biological Target	Biological Activity	Reference
Peptides	*Aspergillus terreus* (No. GX7-3B)	*Bruguiera gymnoihiza* (Linn.) Savigny	Beauvericin (**136**)	AChE inhibitory activity	IC_{50}, 3.09 μM	36
	Colletotrichum gloeosporioides JS419	*Suaeda japonica* Makino	Colletotrichamide A (**137**)	Neuroprotection glutamate-mediated HT22 cells injury	Inactive at 100μM	42
			Colletotrichamide B (**138**)			
			Colletotrichamide C (**139**)		Cell viability of 100% at 100 μM	
			Colletotrichamide D (**140**)		Inactive at 100μM	
			Colletotrichamide E (**141**)			
	Bipolaris sorokiniana LK12	*Rhazya stricta*	BZR-cotoxin I (**142**)	AChE, lipid peroxidation and urease inhibitory activities	Moderate activities	43
			BZR-cotoxin IV (**143**)			
	Cryptosporiopsis sp	*Viburnum tinus*	Cryptosporioptide (**144**)	Lipoxygenase enzyme inhibitory activity	IC_{50}, 49.15 ± 0.17 μM	44

IC_{50}, half maximal effective concentration.
