# Supplementary material for: Anti-Alzheimer’s Natural Products Derived from Plant Endophytic Fungi
Source: Molecules. 2023 Feb 28;28(5):2259. doi: 10.3390/molecules28052259 (PMC10005758; doi:10.3390/molecules28052259)
Supplement: Supplementary file 1 [file molecules-28-02259-s001.zip › Table S3. Polyketides from endophytic fungi and their biological activities, metabolite class, fungus, host plant(s), reference.pdf]

Table S3. Polyketides from endophytic fungi and their biological activities, metabolite class, fungus, host plant(s), reference.

Metabolite Class	Fungus	Host Plant(s)	Compounds Isolated	Biological Target	Biological Activity	Reference
Simple pyranones	*Aspergillus versicolor* Y10	*Huperzia serrata*	Avertoxin A (**145**)	AChE inhibitory activity	Inactive	45
			Avertoxin B (**146**)		IC_{50}, 0.6 μM	
			Avertoxin C (**147**)		Inactive	
			Avertoxin D (**148**)			
			Asteltoxin (**149**)			
	Xylaria sp. HNWSW-2	*Xylocarpus granatum*	Astropyrone (**150**)	AChE inhibitory activity	Inhibition rates of 10.4% at 50 μg/mL	46
	Bipolaris sorokiniana LK12	*Rhazya stricta*	Bipolarisenol (**151**)	AChE inhibitory activity	IC_{50}, 67.23 ± 5.12 μg/mL	47
	Botryosphaeria dothidea KJ-1	*Melia azedarach* L	Pycnophorin (**152**)	DPPH free radical-scavenging	Scavenging rate of 22.5% at 50 μM	34
	Chaetomium globosum	*Panax notoginseng*	Chaetomugilin A (**153**)	DPPH free radical-scavenging	EC_{50} > 100 μg/mL	17
			Chaetomugilin D (**154**)			
Benzopyrones	*Xylomelasma* sp. Samif07	*Salvia miltiorrhiza Bunge*	Hydroxylchromone (**155**)	Hydroxyl radical-scavenging activity	Inactive	48
			6-Hydroxymethyleugenin (**156**)			
			6-Methoxymethyleugenin (**157**)			
			Chaetoquadrin D (**158**)			
			Isoeugenitol (**159**)			
			Diaporthin (**160**)		EC_{50}, 15.1 μg/mL	
			8-Hydroxy-6-methoxy-3-methylisocoumarin (**161**)		Inactive	
			6-Methoxymellein (**162**)			

	Phaeosphaeria sp. LF5	*Huperzia. serrata*	(*R*)-6-Hydroxymellein (**163**)	AChE inhibitory activity	IC$_{50}$>100μM	49
			6,8-Dihydroxy-3-(10*R*, 20*R*-Dihydroxypropyl)-isocoumarin (**164)**			
			6-Dydroxy-8-methoxy-3-methylisocoumarin (**165**)			
			De-O-methyldiaporthin (166)		IC$_{50}$, 21.18 μM	
	Penicillium sp.2/ *Hyalodendriella* sp. Ponipodef 12	*Alibertia macrophylla* (Rubiaceae)/ *Populus deltoides* Marsh × *P. nigra* L. L	4-Dydroxymellein (**167**)	AChE inhibitory activity	MIC, 30 μg	25, 50
	Penicillium sp.2	*Alibertia macrophylla* (Rubiaceae)	8-Methoxymellein (**168**)		MIC ＞ 100 μg	25
			5-Hydroxymellein (**169**)			
	Hyalodendriella sp. Ponipodef 12	'Neva' of *Populus deltoides* Marsh × *P. nigra* L. L	Palmariol B (**170**)	AChE inhibitory activity	IC$_{50}$: 115.31, 135.52, and 10.3.7 μg/mL, respectively	50, 51
			Alternariol 9-methyl ether (**171**)			
			Botrallin (**172**)			50, 51,52
			Hyalodendriol A (**173**)		IC$_{50}$ ＞ 200 μg/mL	50, 51
			Hyalodendriol B(**174**)		IC$_{50}$, 21.1 μg/mL	
			Hyalodendriol C(**175**)		IC$_{50}$ ＞ 200 μg/mL	
			Rhizopycnin D (**176**)			
			Penicilliumolide D (**177**)			
			TMC-264 (**178**)		IC$_{50}$: 89.7 and 49.6 μg/mL, respectively	
			Penicilliumolide B (**179**)			
			Alternariol (**180**)		IC$_{50}$ ＞ 200μg/mL	
			Graphislactone A (**181**)			

Microsphaeropsis olivacea	*Pilgerodendron uviferum* (D. Don) Florin ("Cipres de las Guaitecas")	Graphislactone A (**182**)<br>Graphislactone A diacetate (**183**)<br>Botrallin diacetate (**184**)	AChE inhibitory activity	IC_{50}, 8.1, 88, and 27μg/mL, respectively	52
Colletotrichum sp. CRI535-02	*Piperornatum*	Monocerin (**185**)	DPPH scavening activity, ORAC antioxidant activity	DPPH, IC_{50} > 324 μM, ORAC, 10.8 ORAC unit	53
		Monocerin demethylated derivative (**186**)		DPPH, IC_{50} = 23.4 μM, ORAC, 11.5 ORAC unit	
		Fusarentin 6,7-dimethyl ether (**187**)		DPPH, IC_{50} > 322 μM, ORAC, 14.4 ORAC unit	
		Fusarentin 6-methyl ether (**188**)		DPPH, IC_{50}=16.4 μM, ORAC, 1.4 ORAC unit	
		Fusarentin derivative (**189**)		DPPH, IC_{50} > 306 μM, ORAC, 1.4 ORAC unit	
		Phthalide (**190**)		DPPH, IC_{50} > 324 μM, ORAC, 2.4 ORAC unit	
Penicillium brefeldianum F4a	*Houttuynia cordata*	Penialidin A (**191**)	$ABTS^+$ scavenging activity	EC_{50}, 14.54 ± 0.46 μM	31
		Penialidin F (**192**)	DPPH and $ABTS^+$ scavenging activity	EC_{50}, 28.42 ± 3.16 and 7.61 ± 0.46μM, respectively	
		Myxotrichin C (**193**)		EC_{50}, 30.07 ± 2.83 and 14.96 ± 2.57μM, respectively	
Phomopsis sp. 33#.	*Rhizophora stylosa*	Phomopsichin A (**194**)<br>Phomopsichin B (**195**)<br>Phomopsichin C (**196**)<br>Phomopsichin D (**197**)	AChE inhibitory activity and DPPH scavenging activity	AChE inhibitory rates: 38.4%, 2.7%, 9.1% and 11.3% at 250 μM, respectively.<br>DPPH inhibitory rates:	54

					18.0%, 25.3%, 52.0% , and 17.0% at 1 mM, respectively	
			Phomoxanthone A (198)	DPPH scavenging activity	Inhibitory rate of 40% at 1mM	
	Penicillium sp. FJ-1	*Acanthus ilicifolius* Linn.	(2*R*,3*S*)-Pinobanksin-3-cinnamate (**199**)	Neuroprotection on corticosterone-damaged PC12 cells	Increasing the viability of cells	55
	Alternaria sp	*Maianthemum bifolium*	Bialternacin A (**200**)	AChE inhibitory activity	Inactive	56
			Bialternacin E (**201**)		IC_{50}, 15.5 μM	
			Bialternacin F (**202**)		Inactive	
Pyranyl derivatives	*Penicillium* sp. JVF17	*Vitex rotundifolia*	Peniaphilone A (**203**)	Neuroprotection on glutamate-induced HT22 cells injuries	Inactive	39
			Peniaphilone B (**204**)		-	
			Peniaphilone C (**205**)		Recovering cell viability more than 80% at 25 and 50 μM	
			Peniaphilone D (**206**)		Inactive	
			Peniaphilone E (**207**)			
			Peniaphilone F (**208**)		Recovering more than 80% at 25 and 50 μM	
			Dechloroisochromophilone III (**209**)		Showing protective effects at 50 μM	
			Peniaphilone G (**210**)		Inactive	
			Peniaphilone H (**211**)			
			Peniaphilone I (**212**)			
			Isochromophilone V (**213**)		Showing protective effects at 100 μM	

	Penicillium chermesinum (ZH4-E2)	*Kandelia candel*	Chermesinone A (**214**)	AChE inhibitory activity	IC_{50}>100 μM	57
			Chermesinone B (**215**)			
			Chermesinone C (**216**)			
	Saccharicola sp.	*Eugenia jambolana*	2,2-Dimethyl-2H-chromene-6-carboxylic acid (**217**)	huAChE-ICER and eeAChE-ICER inhibition activities	Inactive	58
			trans-3,4-Dihydro-3,4-dihydroxy-anofinic acid (**218**)		IC_{50}, 0.037 ± 0.01 and 0.026 ± 0.005 mg/mL	
Quinones	*Colletotrichum* sp. JS-0367	*Morus alba* (mulberry)	1,3-Dihydroxy-2,8-dimethoxy-6-methylanthraquinone (**219**)	Neuroprotection on glutamate-induced HT22 cell death	Inactive	59
			1-Hydroxy-2,3,8-trimethoxy-6-methylanthraquinone (**220**)			
			1,2-Dihydroxy-3,8-dimethoxy-6-methylanthraquinone (**221**)			
			Evariquinone (**222**)		Strong protective effects	
	Epicoccum nigrum	*Entadaabyssinica Steud.* ex A. Rich., Fabaceae	Quinizarin (**223**)	ABTS and DPPH scavenging activities	IC_{50}, 10.86 and 11.36 μg/mL, respectively	30
	Chaetomium sp. YMF432	*Huperzia serrata* (Thunb. ex Murray) Trev	1-Omethylemodin (**224**)	AChE inhibitory activity	IC_{50}, 37.7 ± 1.5 and 37.0 ± 2.9 μM, respectively	60
			5-Methoxy-2-methyl-3-tricosyl-1,4-benzoquinone (**225**)			
	Chaetomium sp.	*Huperzia serrata* (Thunb.	Isosclerone (**226**)	AChE inhibitory	AChE inhibition rate less	29,60

	YMF432/ *Alternaria alternate*	ex Murray) Trev/ *Psidium littorale* Raddi.		activity ,neuroprotection for glutamate induced-PC12 cells injury	than 10% at 100 μg/mL, improving cell viabilities from 65.9 ± 3.9% to 74.6 ± 4.0% with 20, 40 and 80 μM	
	Aspergillus terreus (No. GX7-3B)	*Bruguiera gymnoihiza* (Linn.) Savigny	Hydroxy-2-[1-hydroxyethyl]-5,7-dimethoxynaphtho[2,3-b] thiophene-4,9-dione (**227**)	AChE inhibitory activity	Inactive	36
			Anhydrojavanicin (**228**)		IC_{50}, 2.01 μM	
			8-O-Methyljavanicin (**229**)		Inactive	
			Botryosphaerone D (**230**)			
			6-Ethyl-5-hydroxy-3,7-dimethoxynaphthoquinone (**231**)			
	Fusarium sp. HP-2	"Qi-Nan" agarwood	3-Demethoxyl-fusarnaphthoquinone B (**232**)	AChE inhibitory activity	Inactive	37
			(2*S*,3*S*,4*S*)-8-Dehydroxy-8-methoxyl-dihydronaphthalenone (**233**)		Inhibition ratio of 11.9% at 50 μM	
	Talaromyces islandicus EN-501	*Laurencia okamurai*	8-Hydroxyconiothyrinone B (**234**)	Antioxidant activities against DPPH and ABTS	IC_{50} for DPPH: 12, 31, 42, 52 and 30 μM, respectively. IC_{50} for ABTS: 19, 34, 31 and 24 μM, respectively.	61
			8,11-Dihy-droxyconiothyrinone B (**235**)			
			4*R*,8-Dihydroxyconiothyrinone B (**236**)			
			4*S*,8-Dihydroxyconiothyrinone B (**237**)			
			4*S*,8-Dihydroxy-10-O-methyldendryol E (**238**)			

	Colletotrichum sp. F168	*Huperzia serrata* Trev	5-Methoxy-2-methyl-3-pentacosylcyclohexa-2,5-diene-1,4-dione (**239**)	AChE inhibitory activity	Inhibition rate of 10.9% at 100 μg/mL	63
Other polyketides	*Colletotrichum gloeosporioides*	*M. champaca*	2(4-Hydroxyphenyl)acetic acid (**240**)	AChE inhibitory activity	Moderate activity at 200 μg	26
			2(2-Hydroxyphenyl)acetic acid (**241**)			
	Penicillium sp.1	*Alibertia macrophylla* K. Schum. (Rubiaceae)	Orcinol (**242**)	AChE inhibitory activity	Moderate AChE inhibitory activity	25
	Bipolaris sorokiniana LK12	*Rhazya stricta*	Sorokiniol (**243**)	AChE inhibitory activity	EC_{50}, 3.402 ± 0.08 μg/mL	43
	Botryosphaeria dothidea KJ-1	*Melia azedarach* L	Altenusin (**244**)	DPPH radical scavenging activity	IC_{50}, 17.6 ± 0.23 and 18.7 ± 0.18 μM, respectively	33
			5′-Methoxy-6-methylbiphenyl-3,4,3′-triol (**245**)			
	Epicoccum nigrum	*E. abyssinica* Steud. ex A. Rich	Parahydroxybenzaldehyde (**246**)	ABTS and DPPH scavenging activities	IC_{50}, 38.43 ± 4.85 and 49.45 ± 6.52 μg/mL	30
	Phomopsis sp. xy21	Thai *Xylocarpus granatum*	Phomopsol B (**247**)	Neuroprotection on corticosterone induced PC12 cells injury	Inactive	38
			3-(2,6-Dihydroxyphenyl)-4-hydroxy6-methylisobenzofuran-1(3H)-one (**248**)		Improving cell viabilities for 96% at 40.0 μM	
	Penicillium chermesinum (ZH4-E2)	*Kandelia candel*	6′-O-Odesmethylterphenyllin (**249**)	AChE inhibitory activity	IC_{50}＞100μM	57
			3-Hydroxy-6′-O-desmethylterphenyllin (**250**)			
			3,3″-Dihydroxy-6′-O-desmethylterphenyllin (**251**)			

		3″-Deoxy-6′-O-desmethylcandidusin B (**252**)		IC_{50}, 7.8 and 5.2 μM, respectively	
		6′-O-desmethylcandidusin B (**253**)			
Chaetomium globosum	*Panax notoginseng*	Flavipin (**254**)	AChE inhibitory activity	Inhibition ratio ＜ 10% at 50 μM	17
		Epicoccone (**255**)	—	—	
		3-Methoxyepicoccone (**256**)	AChE inhibitory activity	Inhibition ratio of 72.6% at 50 μM	
		Epicoccolide A (**257**)		Inhibition ratio ＜ 10% at 50 μM	
		Epicoccolide B (**258**)		IC_{50}, 5.55 μM	
Microsphaeropsis olivacea	*Pilgerodendron uviferum* (D. Don) Florin	Butyrolactone I (**259**)	AChE inhibitory activity	IC_{50} ＞ 100 μg/mL	52
		Ulocladol diacetate (**260**)		IC_{50}, 83 and 37 μg/mL, respectively	
		Ulocladol triacetate (**261**)			
		2,5-Diacetylphenol (**262**)	–	–	
		7-Hydroxy-2,4dimethyl-3(2*H*)-benzofuranone (**263**)			
		Enalin[2,7-dihydroxy-2,4-dimethyl-3(2*H*)-benzofuranone (**264**)	AChE inhibitory activity	IC_{50}, 89 μg/mL	
Corynespora cassiicola L36	*Lindenbergi philippensis* (Cham.) Benth	Corynesidone A (**265**)	DPPH scavenging activity	IC_{50} ＞ 250 μM	63
		Corynesidone B (**266**)		IC_{50}, 22.4 and 182.4 μM, respectively	
		Corynether A (**267**)			
		A diaryl ether (**268**)	MPP^+-induced oxidative damage in PC12 cells	Improving cell viability	63, 64
Penicillium citrinum	*Bruguiera gymnorrhiza*	(*Z*)-7,40-dimethoxy-6-hydroxy-a	MPP^+-induced oxidative	Improving cell viability and	64

		urone-4-O-b-glucopyranoside (**269**)	damage in PC12 cells	mitochondrial membrane potential, inhibiting caspase-3 and caspase-9 expression and reducing DNA fragments formation	
		(1*S*,3*R*,4*S*)-1-(40-hydroxyl-phenyl)-3,4-dihydro-3,4,5-trimethyl-1*H*-2-benzopyran-6,8-diol (**270**)		Neuroprotective activity	
Pestalotiopsis microspora	*Terminalia morobensis*	Isopestacin (**271**)	Hydroxyl free radical scavenging activity	Scavenging $OH^{\cdot}$ at 0.22 mM	65
Cochliobolus kusanoi	*Nerium oleander* L	Oosporein (**272**)	DPPH scavenging activity	50% scavenging activity at 0.194 mM	66
Sporothrix sp. (#4335)		Sporothrin A (**273**)	AChE inhibitory activity	IC_{50}, 1.05 μM	67, 68
		Sporothrin B (**274**)	–	–	
		Sporothrin C (**275**)			
		Sporothrin C (**276**)			
		1-Hydroxy 8-Methoxy-naphthalene (**277**)	AChE inhibitory activity	Inactive	67
		1,8-Dimethoxy-naphthalene (**278**)			
Alternaria sp	*Maianthemum bifolium*	Bialternacin B (**279**)	AChE inhibitory activity	Inactive	56
		Bialternacin C (**280**)			
		Bialternacin D (**281**)		IC_{50}, 68.3μM	
Phomopsis sp. NXZ-05	*Camptotheca acuminata* DECNE. (Nyssaceae)	8-O-acetylmultiplolide A (**282**)	AChE inhibitory activity	IC_{50}, 1.19 mg/mL	69
		8-O-acetyl-5,6-dihydro-5,6-epoxymultiplolide A (**283**)		IC_{50} > 10 mg/mL	

		5,6-Dihydro-5,6-epoxymultiplolide A (**284**)			
		3,4-Deoxy-3,4-didehydromul-tiplolide A (285)	–	–	
		(4*E*)-6,7,9-Trihydroxydec-4-enoic acid (**286**)			
		Methyl (4*E*)-6,7,9-trihydroxydec-4-enoate (**287**)			
		Multiplolide A (**288**)	AChE inhibitory activity	IC_{50} > 10 mg/mL	
Cladosporium cladosporioides MA-299	*Bruguiera gymnorrhiza*	5*R*-Hydroxyrecifeiolide (**289**)	AChE inhibitory activity	IC_{50} > 50 μM	70
		5*S*-Hydroxyrecifeiolide (**290**)			
		ent-Cladospolide F (**291**)			
		Cladospolide G (**292**)			
		Cladospolide H (**293**)			
		iso-Cladospolide B (**294**)			
		Pandangolide 1 (**295**)		IC_{50}, 40.26 μM.	
Aspergillus flavus	*Corallina officinalis*	(8*E*,12*Z*)-10,11-Dihydroxyoctadeca-8,12-dienoic acid (**296**)	AChE inhibitory activity	Inhibitory rate of 10.3% at 100 μg/mL	71
Curvularia sp. T12	*Rauwolfia macrophylla*	2′-Deoxyribolactone (**297**)	AChE inhibitory activity	IC_{50}, 1.93 and 1.54 μM, respectively	72
		Hexylitaconic acid (**298**)			
Talaromyces aurantiacus		Talaromycin A (**299**)	AChE inhibitory activity	IC_{50}, 12.63 μM	73
		Talaromycin B (**300**)		Inactive	
Curvularia sp.G6-32	*Sapindus sap-onaria* L	E-G6-32 (**301**)	AChE inhibitory activity, DPPH and ABTS scavenging activities	IC_{50} ＞ 350 μg/mL for AChE inhibition, inhibition values of 22.5% to the DPPH and	74

				62.7% to the ABTS	
Daldinia sp.TJ403-LS1	*Anoectochilus roxburghii*	Daldiniol A (**302**)	BChE inhibitory activity	IC_{50} ＞ 200 μM	75
		Daldiniol B (**303**)			
		Daldiniol C (**304**)			
		Daldiniol D (**305**)			
		4-Hydroxy-3-(3-methylbut-3-en-l-ynyl)benzyl alcohol (**306**)		IC_{50}, 6.93 ± 0.71 and 16.00 ± 0.30 μM, respectively	
		Methoxy-3-(3-methylbut-3-enl-ynyl)benzyl alcohol (**307**)			
		Daldiniol E (**308**)		IC_{50} ＞ 200μM	
		Daldiniol F (**309**)		IC_{50}, 23.33 ± 0.55 and 15.53 ± 0.39 μM, respectively	
		Daldiniol G (**310**)			
Saccharicola sp	*Eugenia jambolana*	Speciosin U (**311**)	huAChE-ICER and eeAChE-ICER inhibitory activity	IC_{50}, 0.076 ± 0.01 and 0.0047 ± 0.0009 mg/mL, respectively	58
		Speciosin V (**312**)		Inactive	
		Speciosin W (**313**)			
		4-Hydroxy-3-(3′-methylbut-3′-en-1′-ynyl)-benzoic acid (**314**)			
		4-Hydroxy-3-prenyl-benzoic acid (**315**)			
Alternaria alternate	*Psidium littorale* Raddi	Alternin A (**316**)	Neuroprotective activity against glutamate-induced PC12 cell death	Improving cell viabilities with the values ranging from 64.7 ± 4.9% to 72.3 ± 4.5 at 20, 40 and 80 μM.	29
		Stemphyperylenol (**317**)		Inactive	
		3(ζ)Hydroxy-octadeca-4(*E*),6(*Z*)-			

		dienoic acid (**318**)			
		E-7,9-diene-11-methenyl palmitic acid (**319**)			
		p-Hydroxybenzonic acid (**320**)			
		Benzoic acid (**321**)			
Trematosphaeria terricola	*Artemisia desertorum*	Trematosphone A (**322**)	Neuroprotective effect against corticosterone-induced injury in PC12 cells	Neuroprotection at 6.25 μM	76
		Trematosphone B (**323**)		Inactive	
Phyllosticta capitalensis	*Loropetalum chinense* var. rubrum	Guignardianone G (324)	Neuroprotective activities on glutamate-induced PC12 cells injury	No neuroprotective activity at 40 μM	77
		Xenofuranone B (**325**)		Weak cytotoxicity at 40 μM	
		Linoleic acid (**326**)		EC_{50}, 33.9 μM	
		2-Hexenoic acid (**327**)		Weak cytotoxicity at 40 μM	
Cochliobolus lunatus SCSIO41401		Phthalide glycerol ether (**328**)	AChE inhibitory activity	IC_{50}, 2.5 ± 0.21 μM	40
Phoma sp. YN02-P-3		Phomeketale A (**329**)	AChE inhibitory activity	IC_{50} ＞ 100 μM	78
		Phomeketale B (**330**)			
		Phomeketale C (**331**)		IC_{50}, 40.0 μM	
		Phomeketale D (**332**)		IC_{50} ＞ 100 μM	
		Phomeketale E (**333**)			
		Phomeketale F (**334**)			
Penicillium sp. sk14JW2P	*Kandelia candel* (L.) DRUCE	13-Hydroxypalitantin (**335**)	AChE inhibitory activity	IC_{50}, 12 ± 0.3 and 79 nM, respectively	79
		(+)-Palitantin (**336**)			
Aspergillus sp. xy02	*Xylocarpus moluccensis*	(7*R*,10*S*)-7,10-Epoxysydonic acid	DPPH scavenging	Inactive	80

		(**337**)	activity		
		(7*S*,10*S*)-7,10-Epoxysydonic acid (**338**)			
		(7*R*,11*S*)-7,12-Epoxysydonic acid (**339**)			
		(7*S*,11*S*)-7,12-Epoxysydonic acid (**340**)			
		7-Deoxy-7,14-didehydro-12-hydroxysydonic acid (**341**)			
		(*Z*)-7-Deoxy-7,8-didehydro-12-hydro-xysydonic acid (**342**)			
		(*E*)-7-Deoxy-7,8-didehydro-12-hydroxysydonic acid (3**4**3)			
		(+)-1-Hydroxyboivinianic acid (**344**)			
		Engyodontiumone I (**345**)			
		(+)-Sydonic acid (**346**)			
		(+)-Hydroxysydonic acid (**347**)			
		(−)-(7*S*)-10-Hy-droxysydonic acid (**348**)		IC_{50}, 72.1 µM	
Phaeosphaeria sp. LF5	*Huperzia serrata*	3-(Hydroxymethyl)-5-methylfuran-2(5*H*)-one (**349**)	AChE inhibitory activity	Inactive at 100 µM	49
		Aspilactonol G (**350**)			
		Aspilactonol H (**351**)			
		Aspilactonol I (**352**)		IC_{50}, 6.26 µM	

		E-Δ2-anhydromevalonic acid (**353**)		Inactive at 100 μM	
co-culture of the *Armillaria* sp. and *Epicoccum* sp. YUD17002		Armilliphatic A (**354**)	AChE inhibitory activity	IC_{50}, 23.85 μM	81
		Armilliphatic B (**355**)		Inactive at 50 μM	
		Armilliphatic C (**356**)			
Chaetoium sp. NF00754	*Pharbitis nil*	1-Oxaspiro chaetospirolactone (**357**)	AChE inhibitory activity	Inactive	82
		Orsellide F (**358**)			
		Orsellide A (**359**)		IC_{50}, 7.34 μM	
		Globosumone B (**360**)		Inactive	
		Globosumone C (**361**)		IC_{50}, 7.67 μM	

"_" not test; IC_{50}, half maximal effective concentration; EC_{50}, half effective concentration; Emax, maximum effect; AChE, acethlcholinesterase; DPPH, 2,2-dipheny-1-picrylhydrazyl; ABTS, 2,2-azino-bis(3-ethylbenzothiazoline-6-sulfonic acid) diammonium salt.
