# Supplementary material for: Anti-Alzheimer’s Natural Products Derived from Plant Endophytic Fungi
Source: Molecules. 2023 Feb 28;28(5):2259. doi: 10.3390/molecules28052259 (PMC10005758; doi:10.3390/molecules28052259)
Supplement: Supplementary file 1 [file molecules-28-02259-s001.zip › Table S4. Terpenes from endophytic fungi and their biological activities, metabolite class, fungus, host plant(s), reference.pdf]

Table S4. Terpenes from endophytic fungi and their biological activities, metabolite class, fungus, host plant(s), reference.

Metabolite Class	Fungus	Host Plant(s)	Compounds Isolated	Biological Target	Biological Activity	Reference
Sesquiterpenoids	*Aspergillus* sp. 085242		Asperterpenol A (**362**)<br>Asperterpenol B (**363**)	AChE inhibitory activity	IC_{50}: 2.3 and 3.0 μM, respectively	83
	Alternaria alternate	*Psidium littorale* Raddi	(1*R*,5*R*,6*R*,7*R*,10*S*)-1,6-Dihroxyeudesm-4(15)-ene (**364**)	Neuroprotection against glutamate induced-PC12 cells injury	Inactive at 40 μM and 80 μM	29
	Paecilomyces sp. TE-540	*Nicotiana tabacum* L	Paecilacadinol A (**365**)<br>Paecilacadinol B (**366**)<br>Ustusol D (**367**)<br>Ustusol E (**368**)	AChE inhibitory activity	Inhibition rates: 27.05%, 34.23%, 27.29%, and 42.35% at 40 μM, respectively	84
			12-Hydroxyalbrassitriol (**369**)<br>2-Hydroxyalbrassitriol (**370**)		IC_{50}: 43.02 ± 6.01 and 35.97 ± 2.12 μM, respectively	
			Deoxyuvidin B (**371**)<br>3β,9α,11-Trihydroxy-6-oxodrim-7-ene (**372**)<br>2α,11-Dihydroxy-6-ox-odrim-7-ene (**373**)<br>Ustusol B (**374**)		Inhibition rates: 19.23%, 33.04%, 17.56%, and 24.24% at 40 μM, respectively	
	Pseudofusicoccum sp. J003	*Sonneratia apetala* Buch.-Ham	Acorenone C (**375**)	AChE inhibitory activity	Inhibition rate of 23.34% at 50 μM	85
	Nemania bipapillata	*Asparagopsis taxiformis-*	(+)-(2*R*,4*S*,5*R*,8*S*)-(**376**)	AChE and BChE	Inhibition rates of 19.9%	86

(AT-05)	*Falkenbergia*	(+)-(2*R*,4*R*,5*R*,8*S*)-4-Deacetyl-5-hydroxy-botryenalol (**377**)<br>(+)-(2*R*,4*S*,5*R*,8*R*)-4-Deacetyl-botryenalol (**378**)<br>(+)-(2*R*,4*R*,8*R*)-(**379**)<br>(+)-(2*R*,4*S*,8S)-(**380**)<br>4β-Acetoxy-9β,10β,15α-trihydroxyprobotrydial (**381**)	inhibitory activity	and 14.1% for **376**, 18.3% and 6.7% for **377**, 21.1% and 5.5% for **378**, 27.7% and 7.3% for **379**, 22.8% and 5.1% for **380**, 19.6% and 3.2% for **381**, respectively	
Xylaria sp. HNWSW-2		Guaidiol (**382**)	AChE inhibitory activity	Inhibition rate of 12.9% at 50 μg/mL	46
Nigrospora oryzae	*Irpex lacteus*	Nigrosirpexin A (**383**)	AChE inhibitory activity	Inhibition rate of 35% at 50 μM	87
Colletotrichum gloeosporioides GT-7	*Uncaria rhynchophylla*	Colletotrichine A (**384**)	AChE inhibitory activity	IC_{50}, 28 μg/mL	88
co-culture of the *Armillaria* sp. and *Epicoccum* sp. YUD17002		Epicoterpene A (**385**)<br>Epicoterpene B (**386**)<br>Epicoterpene C (**387**)<br>Epicoterpene D (**388**)<br>Epicoterpene E (**389**)	AChE inhibitory activity	Inactive at 50 μM	47
Phomopsis sp. TJ507A	*Phyllanthus glaucus*	Phomophyllin A (**390**)<br>Phomophyllin B (**391**)<br>Phomophyllin C (**392**)<br>Phomophyllin D (**393**)<br>Phomophyllin E (**394**)<br>Phomophyllin F (**395**)	BACE1 inhibitory activity	Inhibition rates of 43%, 35%, 20%, 40%, 35%, 40%, and 20% at 40 μM, respectively	89

			Phomophyllin G (**396**)			
			Phomophyllin H (**397**)		Inactive	
			Phomophyllin I (**398**)		Inhibition rate about 40% at 40 μM.	
			Phomophyllin J (**399**)		Inactive	
			Phomophyllin K (**400**)			
			Phomophyllin L (**401**)		Inhibition rates about 3%, 5%, 5%, 40%, 2%, 2%, and 43% at 40 μM, respectively	
			Phomophyllin M (**402**)			
			Phomophyllin N (**403**)			
			Granulone B (**404**)			
			Radulone B (**405**)			
			2-(2,2,4,6-Tetramethylindan-5-yl)ethanol (**406**)			
			Pterosin Z (**407**)			
			Onitin (**408**)			
			Dehydrobotrydienol (**409**)		Inactive	
			7-Hydroxy-10oxodehydrodihyd robotrydial (**410**)		Inhibition rate about 42% at 40 μM.	
	Colletotrichum gloeosporioides GT-7	*U. rhynchophylla*	Colletotrichine B (**411**)	AChE inhibitory activity	IC_{50}, 38.0 μg/mL	90
	Colletotrichum sp. SCSIO KcB3-2	*Kandelia candel*	Bisabolanoic acid A (**412**)	AChE inhibitory activity	IC_{50}, 2.2 μM	91
Meroterpenoids	*Penicillium* sp. sk5GW1L	*Kandelia candel*	Arigsugacin I (**413**)	AChE inhibitory activity	IC_{50}: 0.64, 0.37, 7.03, 38.23, 53.39, 3.03, 0.23, and 0.028 μM, respectively	92, 93
			Arigsugacin F (**414**)			
			Territrem B (**415**)			

			3-Epiarigsugacin E (**416**)			
			Arisugacin D (**417**)			
			Arisugacin B (**418**)			
			Territrem C (**419**)			
			Terreulactone C (**420**)			
	Aspergillus terreus Thom	*Tripterygium wilfordii* Hook. f. (Celastraceae)	Spiroterreusnoid A (**421**)	BACE1 and AChE inhibitory activities	IC_{50}: 5.86 and 22.18 μM for **421**, 25.55 and 27.36 μM for **422**, 21.24 and 23.87 μM for **423**, 24.98 and 26.85 μM for **424**, 27.16 and 32.51 μM for **425**, 25.36 and 31.33 μM for **426**, respectively	94
			Spiroterreusnoid B (**422**)			
			Spiroterreusnoid C (**423**)			
			Spiroterreusnoid D (**424**)			
			Spiroterreusnoid E (**425**)			
			Spiroterreusnoid F (**426**)			
	Aspergillus 16-5c	*Sonneratia apetala*	2-Hydro-acetoxydehydroaustin (**427**)	AChE inhibitory activity	$IC_{50} > 50$ μM	95
			11-Acetoxyisoaustinone (**428**)			
			Isoaustinol (**429**)		IC_{50}, 2.50 μM	
			Austin (**430**)		$IC_{50} > 50$ μM	
			Austinol (**431**)			
			Acetoxydehydroaustin (**432**)			
			Dehydroaustin (**433**)		IC_{50}, 0.40 and 3.00 μM, respectively	
			Dehydroaustinol (**434**)			
			Preaustinoid A2 (**435**)		$IC_{50} > 50$ μM	
			1,2-Dihydro-acetoxydehydroaustin B (**436**)			
Diterpenoids	*Penicillium chrysogenum* MT-12	*Huperzia serrata*	Penicichrysogene A (**437**)	AChE and BChE inhibitory activities	Inactive at 100 μM	96

			Penicichryso-gene B (**438**)			
	Aspergillus sp. YXf3	*Ginkgo biloba*	Aspergiloid I (**439**)	Anti-oxidant and AChE inhibitory activities	Inactive at 50 μg/mL	97

IC_{50}, half maximal effective concentration; AChE, acethlcholinesterase; BChE, Butyrylcholinesterase.
