# Supplementary material for: Anti-Alzheimer’s Natural Products Derived from Plant Endophytic Fungi
Source: Molecules. 2023 Feb 28;28(5):2259. doi: 10.3390/molecules28052259 (PMC10005758; doi:10.3390/molecules28052259)
Supplement: Supplementary file 1 [file molecules-28-02259-s001.zip › Table S5. Steroids from endophytic fungi and their biological activities, metabolite class, fungus, host plant(s), reference.pdf]

Table S5. Steroids from endophytic fungi and their biological activities, metabolite class, fungus, host plant(s), reference.

Metabolite Class	Fungus	Host Plant(s)	Compounds Isolated	Biological Target	Biological Activity	Reference
Steroids	*Aspergillus oryzae*	*Heterosiphonia* japonica	Asporyergosterol (**440**)	AChE inhibitory activity	Inhibition rate of 14.0% at 100 μg/mL	98
	Aspergillus oryzae/ *Alternaria alternate*	*Heterosiphonia japonica*/ *Psidium littorale*	(22*E*,24*R*)-Ergosta-4,6,8(14),22-tetraen-3-one (**441**)	AChE inhibitory activity	Inhibition rate of 19.8 % at 100 μg/mL	98, 29
	Aspergillus oryzae	*Heterosiphonia japonica*	(22*E*,24*R*)-3β-Hydroxyergosta-5,8,22-trien-7-one (**442**)	AChE inhibitory activity	Inhibition rate of 7.2% at 100 μg/mL	98
	Aspergillus oryzae/ *Chaetomium* sp. M453/ *Talaromyces* sp. SCNU-F0041	*Heterosiphonia japonica*/ *Huperzia* serrate (Thunb. ex Murry) Trev/ *Kandelia*	(22*E*,24*R*)-Ergosta-7,22-dien-3β,5α,6β-triol (**443**)	AChE inhibitory activity	Inhibition rate of 0.4% at 100 μg/mL	98, 100
	Aspergillus oryzae	*Heterosiphonia japonica*	(22E,24R)-5α,8α-Epidioxyergosta-6,22-dien-3β-ol (**444**)	AChE inhibitory activity	Inhibition rate of 8.1% at 100 μg/mL	98
	Aspergillus flavus cf-5	*Corallina officinalis*	3β,4α-Dihydroxy26-methoxyergosta-7,24(28)-dien-6-one (**445**)	AChE inhibitory activity	Inhibition rate of 5.5% at 100 μg/mL	71
			Episterol (**446**)	_	_	
			(22*E*,24*R*)-Ergosta7,22-dien-3β,5α,6α-triol (**447**)			
	Aspergillus flavus cf-5/ *Phyllosticta capitalensis*	*Corallina officinalis*/ *Huperzia serrate* (Thunb. ex Murry) Trev/ *Loropetalum chinense var.* rubrum	(22*E*,24*R*)-Ergosta-5,22-dien-3β-ol (**448**)	_	_	71, 77
	Chaetomium sp.	*Huperzia serrate* (Thunb. ex	Neocyclocitrinol E (**439**)	_	_	99

M453	Murry) Trev	Neocyclocitrinol F (**450**)			
		Neocyclocitrinol G (**451**)	AChE inhibitory activity	Inactive	
		3β-Hydroxy-5,9-epoxy-(22*E*,24*R*)-ergosta-7,22-dien-6-one (**452**)		Weak activity at 50 μM	
		**453**		Inactive	
Chaetomium sp. M453/ *Phyllosticta capitalensis*	*Huperzia serrate* (Thunb. ex Murry) Trev/ *Loropetalum chinense* var. rubrum	**454**	AChE inhibitory activity	Inactive	99, 77
Chaetomium sp. M453	*Huperzia serrate* (Thunb. ex Murry) Trev	**455**	AChE inhibitory activity	Inactive	99
Chaetomium sp. YMF432	*Huperzia serrata* (Thunb. ex Murray) Trev	(3β,5*α*,6*α*,22E)-3-Hydroxy-5,6-epoxy7-one-8(14),22-Dien-ergosta (**456**)	AChE inhibitory activity	IC_{50}, 67.8 ± 1.7 μM	60
		β-Sitostenone (**457**)		Inhibition rate ＜ 10% at 100 μg/mL	
		β-Sitosterol (**458**)			
Aspergillus terreus (No. GX7-3B)	*Bruguiera gymnoihiza* (Linn.) Savigny	3β,5α-Dihydroxy-(22*E*,24*R*)-ergosta-7,22-dien-6-one (**459**)	AChE inhibitory activity	Inactive	36
		3β,5α,14α-Trihydroxy-(22*E*,24*R*)-ergosta-7, 22-dien-6-one (**460**)			
		NGA0187 (**461**)		IC_{50}, 1.89 μM	
Curvularia. Sp/ *Talaromyces* sp. SCNU-F0041	*Rauwolfia macrophylla*/ *Kandelia*	Ergosterol (**462**)	AChE inhibitory activity	IC_{50}, 1.52 μM	72

Alternaria alternate	*Psidium littorale* Raddi	(17*R*)-4-Hydroxy-17-methylincisterol (**463**)	Neuroprotective activity of glutamate induced-PC12 cells	Inactive at 40 and 80 μM	29
Colletotrichum sp. F168	*Huperzia serrata* Trev	Ergosta-7,22-dien-5,9-epoxy-(22*E*,24*R*)-6-one-3-yl acetate (**464**)	AChE inhibitory activity	Inhibition rate of 18.2% at 100 μg/mL	62
Talaromyces sp. SCNU-F0041	*Kandelia*	Cyclosecosteroid A (**465**)	AChE inhibitory activity	IC_{50}, 46 μM	100
		(22*E*,24*R*)-5α,8α-Epidioxyergosta-6,22-dien-3β-ol (**466**)	–	–	
Phyllosticta capitalensis	*Loropetalum chinense* var. rubrum	Citreoanthrasteroid A (**467**)	Neuroprotective effect in PC12 cells injury	EC_{50}, 24.2 μM	77
		Chaxine C (**468**)		Inactive at 40 μM	

"_" not test. IC_{50}, half maximal effective concentration; Emax, maximum effect; AChE, acethlcholinesterase.
